# Evaluating 2D and 3D graph encoders for infrared-based organic structure verification

**DOI:** 10.1039/d6dd00281a

**Published:** 2026-08-19

**Authors:** J. Benji Rowlands, Richard J. Lewis, Peter Howe, Jonathan M. Goodman

**Affiliations:** a Yusuf Hamied Department of Chemistry, University of Cambridge Cambridge UK jmg11@cam.ac.uk; b Medicinal Chemistry, R&D, AstraZeneca Gothenburg Sweden; c Oncology Chemistry, AstraZeneca Cambridge UK

## Abstract

Infrared (IR) spectrum prediction is typically judged by global spectral error, but the practical task in analytical chemistry is structure verification: given a spectrum, can predicted spectra rank the correct structure highly among many candidates? We train Phase-Aware Spectrum Generative Network (PASGeN) models to predict experimental IR spectra and evaluate their predictions using both spectral similarity and retrieval metrics. Using 13 568 experimental IR spectra in various phases, we train models with a 2D message-passing neural network (MPNN) and a 3D equivariant encoder (PaiNN), and fine-tune the 3D model on inexpensive force-field geometries to reduce inference cost. Across large-scale retrieval tests, the 3D encoder significantly outperforms the 2D baseline (73.9% *vs.* 62.9% Top-1 accuracy). On this benchmark, both learned models achieve higher retrieval accuracy than the B3LYP/6-31G(d) and GFN2-xTB protocols evaluated here, which use gas-phase harmonic spectra with simple broadening. Descriptor analysis suggests that ring-rich, geometrically complex molecules benefit most from the inclusion of 3D information. By evaluating IR prediction models on a structure verification task and demonstrating that lightweight 3D geometries provide a measurable advantage, this work establishes a practical and scalable route to IR-based structure confirmation that outperforms reference physics-based baselines at a fraction of the computational cost.

## Abstract 2

The practical task for IR spectroscopy in analytical chemistry is structure verification: given a spectrum, can predicted spectra rank the correct structure highly among many candidates?

## Graphical abstract

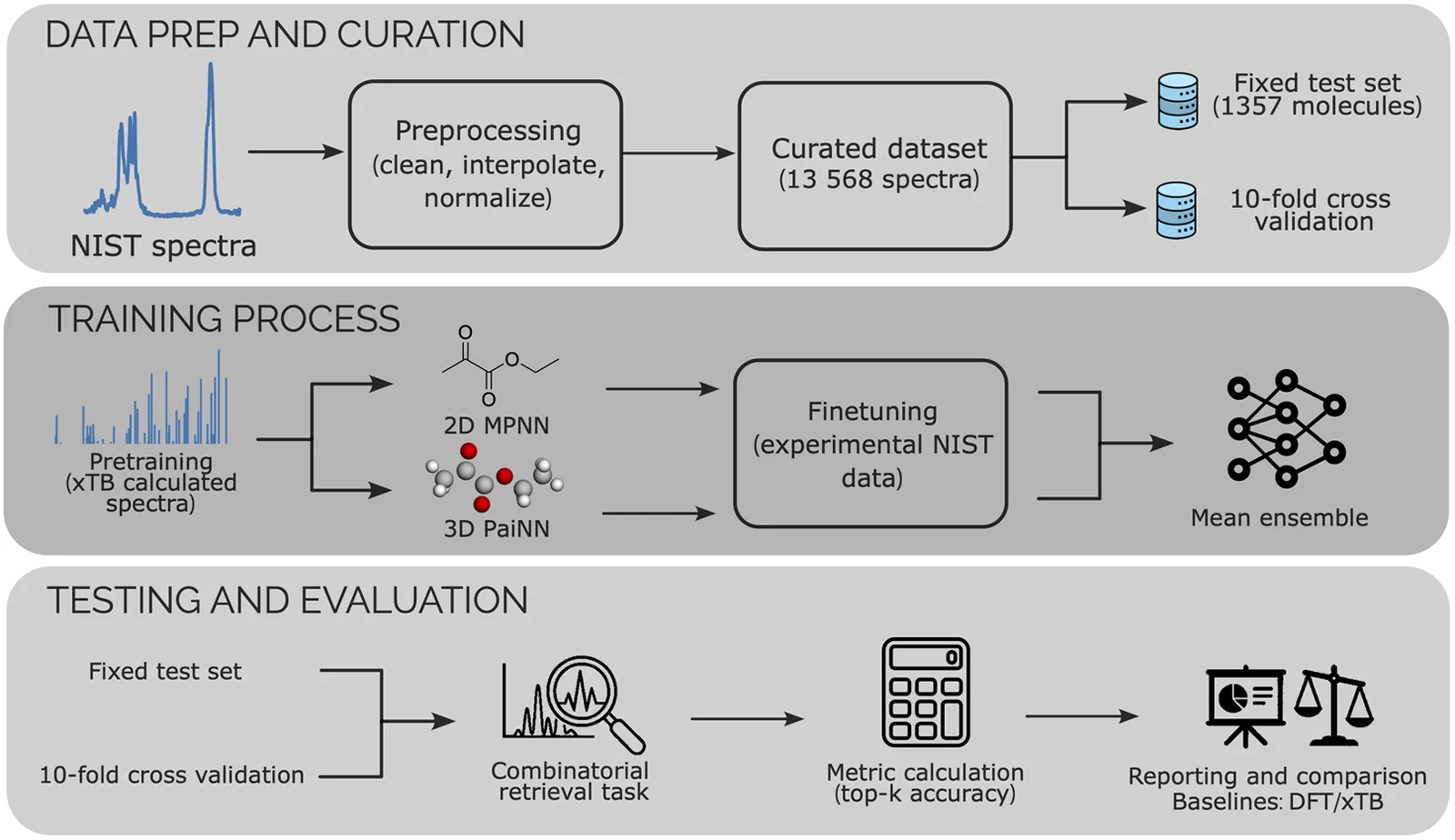

## 1 Introduction

Infrared (IR) spectroscopy is a rapid, accessible, and cost-effective technique for molecular characterisation. Its use has traditionally been limited to identifying diagnostic functional groups, but the entire IR spectrum encodes rich structural information,^1,2^ with the low-wavenumber “fingerprint region” widely used for structure confirmation against experimental databases.^3^

Recent machine-learning (ML) advances have revitalised interest in full-spectrum IR. Alberts *et al.* introduced a transformer model trained on simulated and experimental data,^4^ later refined to set the current benchmark for IR-based *de novo* structure generation.^5^

An alternative to structure generation is spectrum prediction: calculate or predict the IR spectrum of a proposed structure and compare it with experiment. The accuracy and efficiency of this step are critical. Density functional theory (DFT) remains widely used,^6–8^ but is costly and limited by the harmonic approximation. Extended tight-binding (xTB) methods are faster,^9–11^ but share the limitation of the harmonic approximation. By contrast, molecular dynamics (MD) based approaches can circumvent the harmonic approximation of such static calculations and capture anharmonicity and more realistic line shapes,^12,13^ though at prohibitive computational cost.

ML models offer a promising alternative, producing broadened spectra directly from structure at negligible cost. Examples in the literature include Chemprop-IR,^14^ trained on calculated and experimental data, and Graphormer-IR,^15^ which modestly improved prediction performance. These, like most IR models,^14–16^ rely on 2D graphs, which are simple to construct and effective across many property-prediction tasks.^17–20^ However, IR spectra do depend on 3D geometry,^21,22^ and these 2D graphs omit geometric information. There is growing evidence that 3D inputs improve model accuracy across various prediction tasks,^17–20^ with practical workflows now making conformer generation and optimisation affordable.^9,23^ Modern 3D graph encoders can exploit this information effectively at scale,^18,19,24,25^ and have been shown to give good performance for IR prediction when trained on a relatively small dataset of around 5000 gas-phase spectra.^26^

Prior benchmarks have typically emphasised global similarity between predicted and experimental spectra.^14,15^ However, in many practical workflows, the relevant task is to distinguish the correct structure from plausible candidates, so retrieval metrics are more aligned with end use. Global spectral similarity remains important as an absolute measure of how well a molecule matches experimental data; accordingly, we assess both spectral similarity and retrieval performance. This contrasts with approaches focusing on direct structure generation from IR spectra,^27^ which lack the interpretability that comes with obtaining a full predicted spectrum. Similarly, prior work on contrastive learning has shown promise in ranking candidates based on experimental IR data,.^28^ However, because it ranks candidates in a latent space rather than producing a predicted spectrum, it does not provide the same direct spectral interpretation of peak positions, intensities, and mismatched regions. Furthermore, similar setups using an explicit retrieval objective would require careful thought about the construction of the candidate set, in order for the approach to remain generalizable, and may result in a trade-off between spectral prediction accuracy and retrieval performance.

In this work, we compare 2D (MPNN^14^) and 3D (PaiNN^25^) encoders, assessing both spectral accuracy and discriminative power. We benchmark against the original PASGeN architecture, Chemprop-IR, a contrastive learning-based method^28^ and specific quantum-chemical methods,^29^ extending retrieval analysis to ML models to compare learned-spectrum retrieval with DFT and GFN2-xTB calculations and to investigate how encoder choice affects verification performance.

Our models extend the PASGeN framework^30^ with 2D and 3D encoders implemented in PyTorch-Geometric,^31^ enabling a comprehensive assessment of IR-based structure verification with both connectivity-based and geometric inputs. PASGeN predicts IR spectra by first encoding the molecular input into a latent representation that captures structural features relevant to vibrational modes and functional-group environments. This encoded representation is then used by the downstream prediction module to generate the corresponding IR spectrum. In this workflow, the encoder architecture is central because it determines how molecular connectivity and local chemical environments are represented before spectral intensities and peak positions are predicted. Full architectural and training details of PASGeN are provided in the original PASGeN paper.^30^Fig. 1 provides an overview of the data curation, model training and evaluation steps completed in this work.

**Fig. 1:**
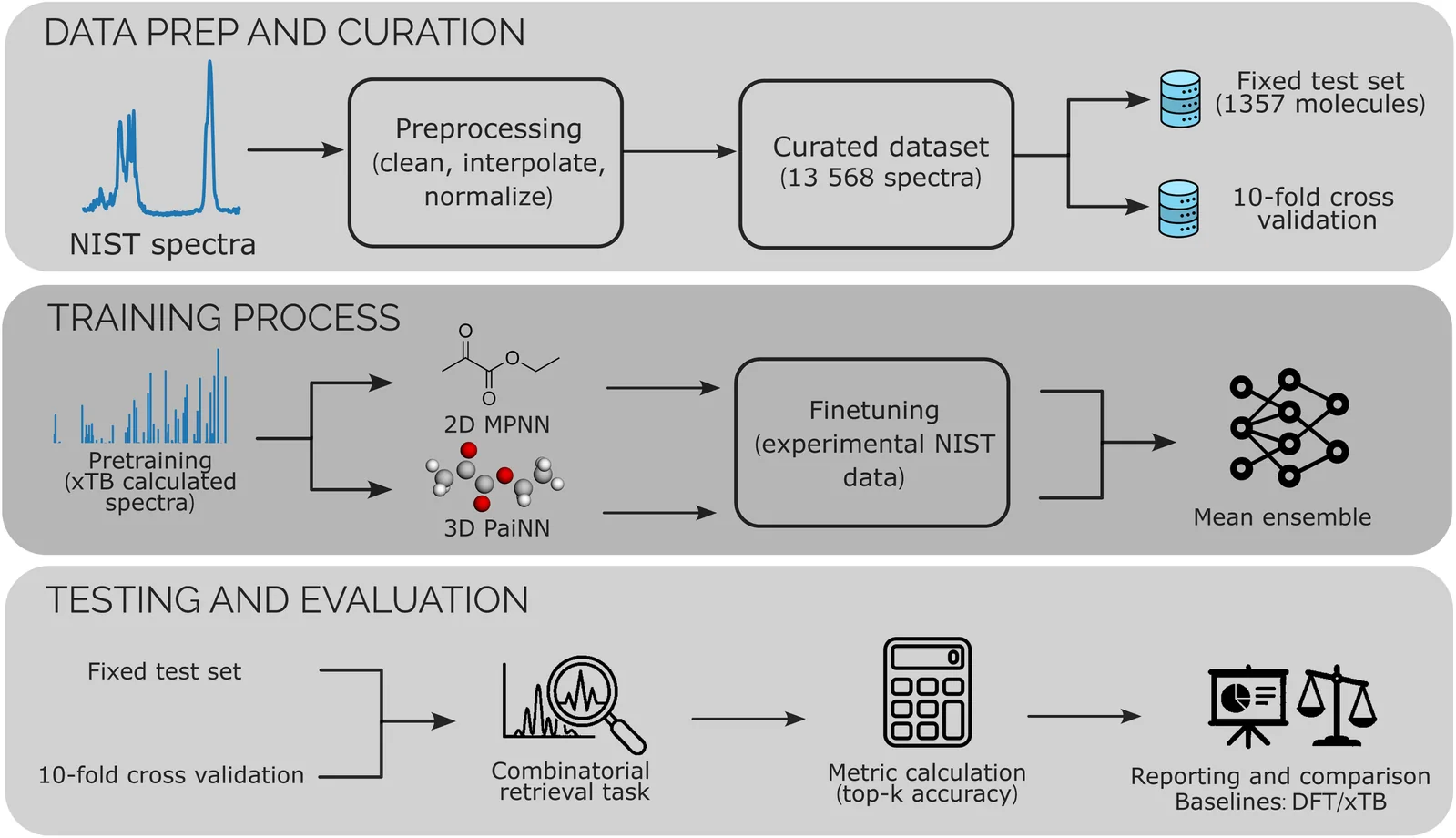
Schematic summarising the data curation, training and testing steps in this work.

## 2 Dataset

### 2.1 Spectra

We downloaded around 16 000 digitised IR spectra from the NIST WebBook^32^ using a BeautifulSoup script,^16^ converted all traces to absorbance, represented them on a wavenumber grid in cm^−1^ and normalised each spectrum to unit intensity; SI Section 1 summarises the full preprocessing pipeline.

### 2.2 Preparation-dependent masking

Sample-preparation solvents introduce strong absorptions, so we zero-mask the corresponding bands before training or evaluation. Full details of the masked ranges for each solvent appear in Table S2.

### 2.3 Molecular structures

We generated 3D conformers for each molecule with RDKit's ETKDG^23^ algorithm and retained up to ten force-field optimised candidates per structure. Dataset curation involved excluding compounds not containing carbon, or containing atoms other than C, H, N, O, P, S, F, Cl, Br, I, B, or Si, and retaining only the organic fragment of salts. Full details are available in the SI (Section 1). After curation, we obtain the final 13 568-spectrum dataset with phase annotations.

### 2.4 Data splits

For all data splits, we use InChIKey-prefix stratification (first 14 characters) to keep structures with the same connectivity together. This prefix is used only as a grouping key for assigning structures to folds and is not supplied to the models. Consequently, stereochemical variants sharing a connectivity are placed in the same fold, preventing leakage between training and test sets; stereochemical information in the molecular inputs is otherwise retained where supported by the encoder. The resulting evaluation therefore tests generalisation to unseen molecular connectivities. We define a fixed 1357-molecule test set on which we performed DFT calculations, and use this fixed test set for comparisons with DFT and xTB baselines. In addition, we perform ten-fold cross validation on the full 13 568-molecule dataset when training and evaluating the neural network models, in order to assess variance across data splits and ensure the robustness of our results.^33^ The curated molecules (13 568 entries with annotated phases) are partitioned into ten disjoint folds. For each fold we train on the other nine folds, reserve 10% of that training portion for validation, and evaluate on the held-out fold. Unless stated otherwise, figures report the fixed-test-set results when physics-based baselines are shown and aggregate the ten cross-validation evaluations when comparing the neural models. The number of held-out spectra in each cross-validation fold is reported in SI Table S7.

## 3 Models

### 3.1 PASGeN backbone

Each model outputs an intensity vector *ŷ*_*i*_ ∈ *ℝ*^*N*_bins_=751^ through the original PASGeN decoder (window size 24, one Transformer block and a lightweight LeakyReLU MLP). Phase (gas/liquid/solid) is embedded in a learned 32-dimensional vector; for all encoders, PASGeN uses this embedding to compute attention weights over atom embeddings (by concatenating each atom embedding with its phase embedding), yielding a state-aware pooled vector that is concatenated with the base molecular embedding before decoding. The phase embedding also conditions the encoders, as described below.

### 3.2 Encoders

The 2D model uses an MPNN encoder over the molecular graph, following Chemprop-IR;^14^ the phase embedding is concatenated to bond features for message passing. The 3D encoder uses PaiNN,^25^ an equivariant message-passing network that combines directional geometric information with atom-wise features. Here, the phase embedding is projected and added to the atom embeddings after edge aggregation. Both encoders yield 128-dimensional embeddings that the decoder maps to *ŷ*_*i*_, and we form a mean ensemble by averaging the two predicted intensity vectors followed by the same normalization as for single models. A comparison of the inputs, featurisations and outputs of all benchmarked methods is provided in SI Table S4.

### 3.3 Conformer augmentation

During training, 3D encoders were provided with one conformer per step sampled from a small (up to 10 conformers) precomputed ensemble per molecule. This improves generalisation to conformational variability while avoiding expensive conformational searches at inference time. For comparison, we also trained with a single fixed conformer. To further reduce the cost of geometry preparation at inference time, we fine-tuned the GFN2-xTB PaiNN checkpoint on MMFF94 (ref. 34 and 35) optimised geometries. The resulting model depends only on inexpensive force-field geometries, allowing rapid inference from a single force-field optimised conformer. All results are reported in the main text for the MMFF fine-tuned model.

## 4 Training details

We optimise all models with AdamW, combine mean-squared error with a negative Pearson correlation term (MSE – *ρ*) on the masked spectra, and rely on early stopping; SI Section 5 provides full training details.

## 5 Contrastive learning baseline

For the contrastive baseline, we followed the architecture/training protocol of Kanakala *et al.*^28^ as closely as possible and trained/evaluated it on the same fixed splits used for the other baselines. Candidate structures were ranked by cosine similarity in the learned joint embedding space. Unlike the PASGeN-based models, this baseline does not output a predicted spectrum and was not conditioned on sample-preparation phase.

## 6 Evaluation metrics

### 6.1 Combinatorial retrieval study

Our primary metric is Top-*k* retrieval accuracy: for each query spectrum we rank the candidate structures by a similarity score (cosine similarity or SIS) and record whether the correct structure appears in the top *k*.

We evaluate several wavenumber windows, including the full 500–3500 cm^−1^ range and the sub-windows used in the main figures. All spectra are interpolated to a uniform 4 cm^−1^ grid over this range (751 bins). Before computing similarities we normalise each spectrum so that its integrated absorbance over the evaluation window equals unity, following Pracht.^11^ We report cosine similarity and spectral information similarity (SIS)^14^ as spectral similarity metrics.

For retrieval, the candidate set for each query comprises the entire held-out test fold, giving approximately *N*^2^ ≈ 1.84 × 10^6^ query–candidate comparisons when *N* ≈ 1357. Top-*k* accuracy is then the fraction of queries whose correct structure appears in the top *k* ranks once all candidates have been scored by cosine similarity or SIS. The workflow is summarised in Fig. 2.

**Fig. 2:**
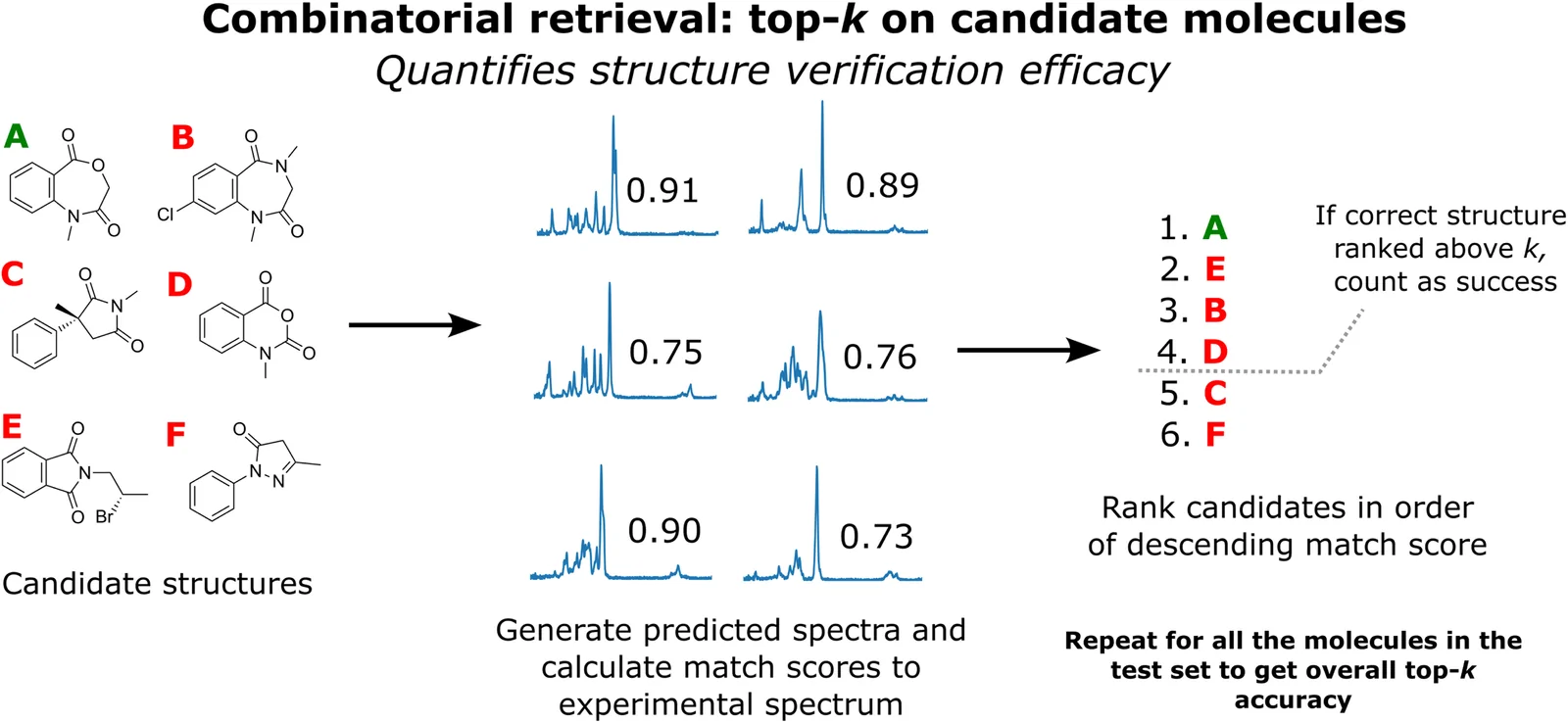
Schematic of the combinatorial retrieval pipeline used for the Top-*k* metrics.

### 6.2 Regression-on-ranks analysis

To understand when 3D helps over 2D, we relate per-query retrieval performance to standard molecular descriptors. We assemble descriptor vectors for every test molecule, remove constant or non-numeric columns, and standardise the remaining features before pruning those whose absolute pairwise correlation exceeds 0.9. For the retrieval targets we consider the reciprocal rank for each model alongside the PaiNN–MPNN difference. We fit partial least squares (PLS) regression models to quantify the linear relationships.^36^ Coefficients and their uncertainty are estimated *via* 5000 bootstrap resamples^37^ (with replacement) to obtain two-sided 95% confidence intervals.

## 7 Results and discussion

### 7.1 Prediction accuracy and retrieval performance

Because the practical question is “does the correct structure sit near the top of the candidate list?”, we centre this section on retrieval metrics and treat global similarity scores as supporting evidence. Fig. 3a shows a heat map of mean cosine similarity score by model and evaluation window; panel b shows Top-1 retrieval accuracy. We focus on the 2D MPNN and MMFF-geometry PaiNN model (referred to as PaiNN for brevity), and include a simple ensemble (mean of the two predictions, using MMFF geometries for the 3D branch). We also include, for additional context, results from DFT (B3LYP/6-31G(d)) and GFN2-xTB calculations. Across windows spanning 500–3500 cm^−1^, we observe that performance varies by region, with the 1000–1800 cm^−1^ range typically easier to predict on average than the full window (higher average cosine similarity). However, retrieval accuracy benefits from using the full spectrum despite lower mean similarity, consistent with more discriminative information being available when all bands are considered. The exception to this rule is that DFT-calculated spectra are most useful using the 500–1800 cm^−1^ window, likely because the high-frequency region is poorly modelled by gas-phase quantum calculations that omit anharmonicity and condensed-phase effects.^38–40^ In particular, experimental peak widths are often poorly reproduced by DFT calculations in this wavenumber range.^41,42^

**Fig. 3:**
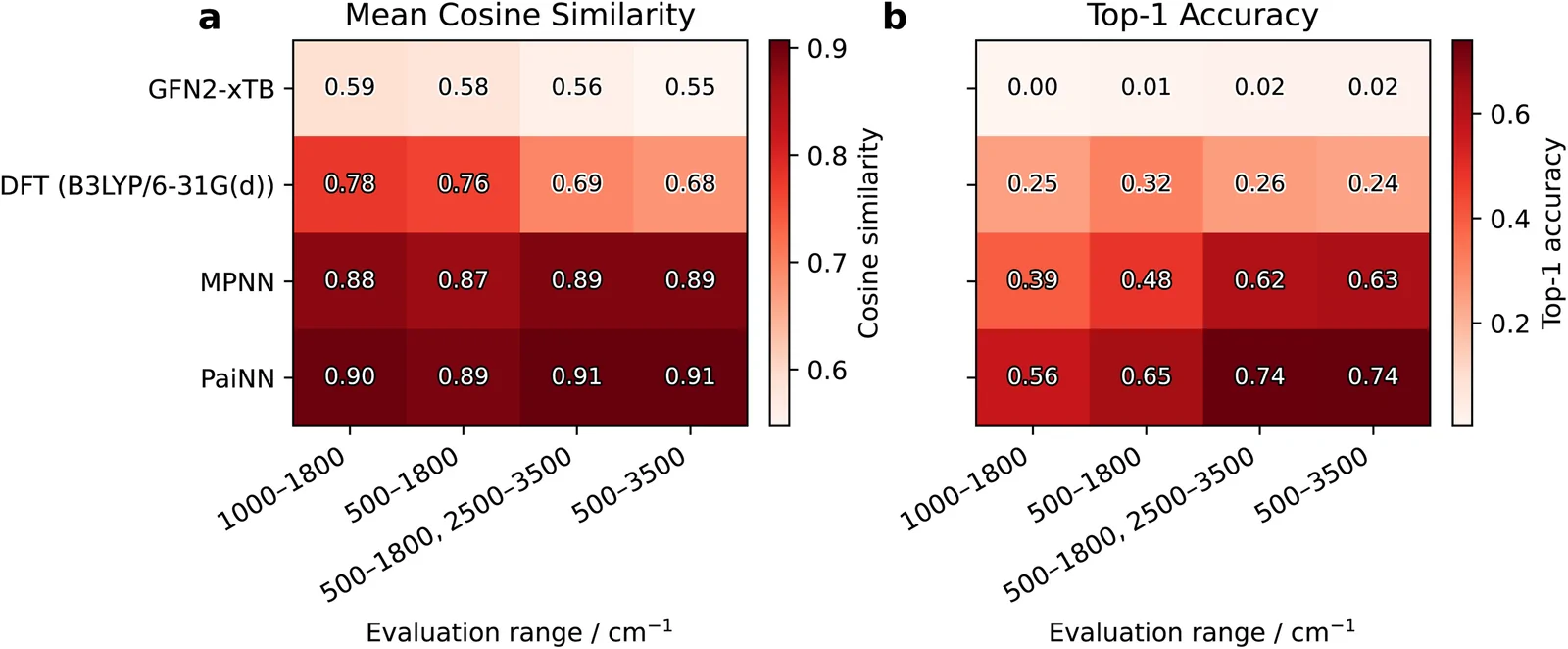
(a) Heat map of mean cosine similarity for each model and wavenumber range. (b) Heat map of Top-1 retrieval accuracy for each model and range.

For the fixed 1357-molecule test set under cosine similarity-based retrieval, the Top-1 success rates inferred from the like-for-like rank tables are 0.629 for MPNN, 0.739 for PaiNN and 0.760 for the PaiNN + MPNN ensemble. In absolute counts this equates to 853, 1003 and 1031 correct top-1 identifications respectively, out of 1357 queries. PaiNN therefore achieves 150 more correct identifications over MPNN, while the ensemble adds another 28 successes beyond the 3D model. Mean retrieval rank follows the same trend (16.217 for MPNN, 10.399 for PaiNN, 9.621 for the ensemble). Table 1 summarises the ten-fold cross-validation Top-1 accuracy and cosine similarity for these models. The original PASGeN baseline is close to, but slightly below, the MPNN model (0.625 *versus* 0.629 Top-1 accuracy on the fixed test set, and 0.598 ± 0.019 *versus* 0.623 ± 0.033 over ten folds). Chemprop-IR, which uses a similar 2D message-passing encoder to the MPNN model and performs better than MPNN. Because this model overall has substantially more trainable parameters, we treat it primarily as a stronger 2D reference model rather than a like-for-like encoder substitution. The performance of Chemprop-IR compared to PaiNN is also somewhat closer than the MPNN *vs.* PaiNN comparison, but the 3D model still outperforms Chemprop-IR (73.9% *vs.* 70.0% Top-1 accuracy on the fixed test set). The contrastive learning-based method performs substantially worse than the other models, although this is likely at least partially due to its architecture not being conditioned on sample preparation phase – this is an obvious area for improvement for such approaches. Fig. 4 shows Top-*k* curves (*i.e.* Top-*k* accuracy as a function of *k*): the left panel corresponds to the fixed 1357-molecule test set (enabling the DFT/xTB comparison) and the right panel aggregates the ten cross-validation folds for the neural models. Additional comparisons to models trained on xTB geometries are included in the SI.

**Table 1:** Summary of performance on the fixed 1357-query benchmark (500–3500 cm^−1^) and the ten-fold cross-validation benchmark. The contrastive learning-based method is scored with embedding cosine; all other rows use masked spectral cosine. The contrastive learning-based method was only evaluated on the fixed test set, so ten-fold entries are shown as “—”

Model	Top-1 (10-fold)	Cosine similarity (10-fold)	Top-1 (fixed test)
MPNN	0.623 ± 0.033	0.888 ± 0.006	0.629
Original PASGeN	0.598 ± 0.019	0.881 ± 0.003	0.625
PaiNN	0.717 ± 0.015	0.904 ± 0.004	0.739
PaiNN + MPNN ensemble	0.740 ± 0.017	0.911 ± 0.004	0.760
Chemprop-IR	0.693 ± 0.011	0.902 ± 0.003	0.700
Contrastive learning baseline^28^	—	—	0.519

**Fig. 4:**
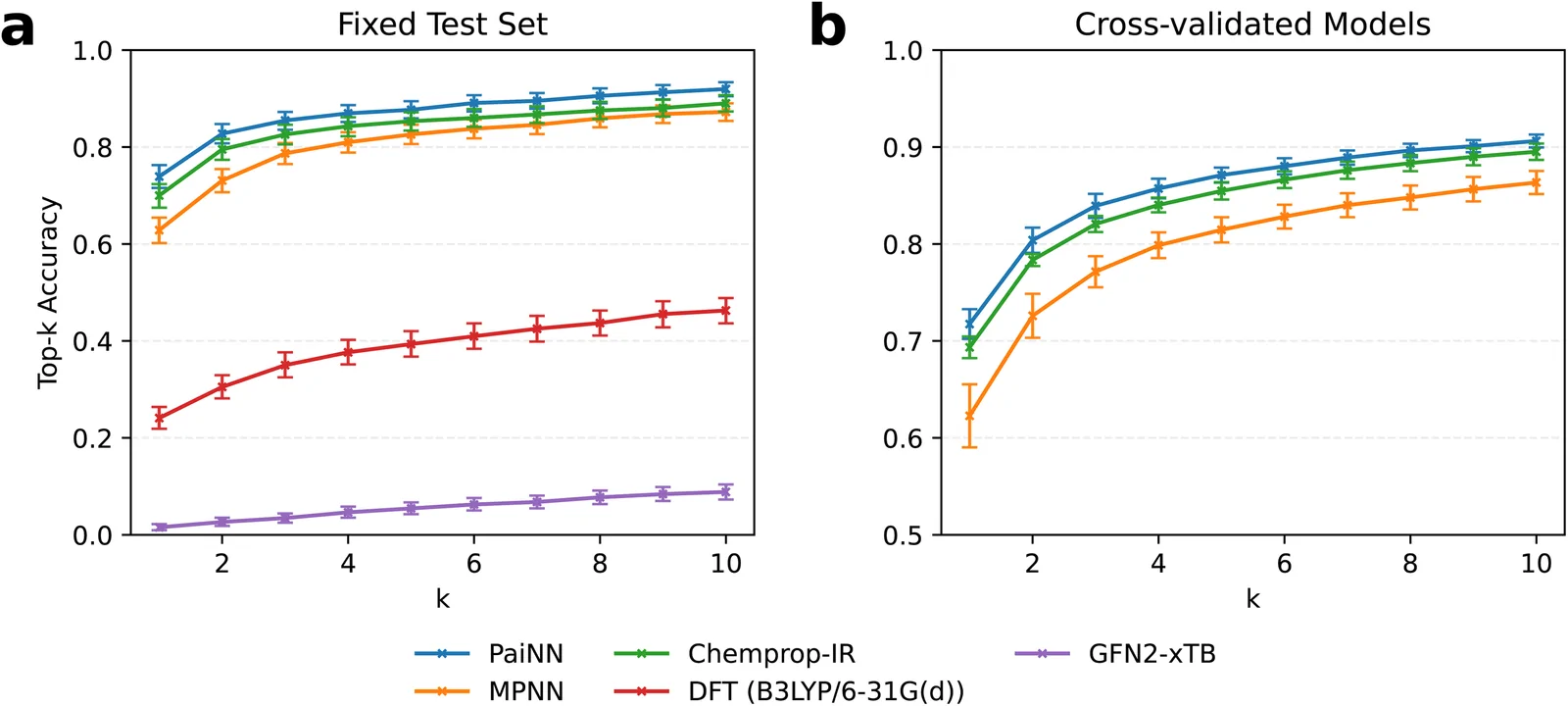
Top-*k* retrieval accuracy curves for the 500–3500 cm^−1^ window. Error bars denote 95% bootstrap confidence intervals over 5000 resamples. (a) Evaluation on the fixed 1357-molecule test set and including the DFT and GFN2-xTB reference calculations. (b) Evaluation across the ten cross-validation folds, only including the neural networks.

Although Top-1 accuracy is the strictest verification metric, Top-*k* accuracy for larger *k* is also practically relevant. In many structure-elucidation workflows, IR would not be used in isolation to make a final assignment, but rather to prioritise a small number of plausible candidates for further inspection using complementary information such as molecular formula, NMR, mass spectrometry, chromatographic behaviour, or synthetic context. In this setting, ranking the correct structure within the top few candidates can still substantially reduce the search space, even when the IR model alone does not place the correct candidate first.^43^

Across the ten cross-validation folds the neural models preserve the same ordering: MPNN achieves 0.623 ± 0.033 Top-1 accuracy, PaiNN reaches 0.717 ± 0.015, and the ensemble gives 0.740 ± 0.017 when averaged over folds (Table 1). Fold-level accuracies for all neural variants, including the xTB geometries, appear in Table S8. These cross-validation runs cover the entire curated dataset (13 568 spectra), substantially reducing variance relative to the fixed hold-out set. Unless explicitly stated otherwise, headline retrieval metrics therefore refer to the aggregated ten-fold evaluations summarised in Table 1.

All pairwise differences in Top-1 accuracy among the learned models are statistically significant: exact McNemar tests^44,45^ on paired query outcomes reject the null of equal accuracy for PaiNN over MPNN, ensemble over MPNN, and ensemble over PaiNN (*p* < 0.001 in all cases). Bootstrap confidence intervals for the Top-1 rates and their pairwise differences are tabulated in the SI (Tables S6 and S9).

For context, we also evaluate specific B3LYP/6-31G(d) and GFN2-xTB reference calculations on this task. On the full 500–3500 cm^−1^ window, the DFT setup achieves only 327 top-1 retrievals (24.1% accuracy), while a faster semiempirical xTB baseline reaches 21 (1.5%). The inclusion of liquid- and solid-phase spectra in our test set makes retrieval challenging for gas-phase quantum calculations, which do not capture matrix and condensed-phase effects and use a rudimentary constant-width Lorentzian line profile to broaden the calculated “stick” spectra. Learned models trained directly on experimental spectra more faithfully reproduce band shapes and relative intensities across phases, enabling stronger verification performance. These calculations provide computationally tractable reference points for retrieval performance and cost; they are not intended to represent the best accuracy attainable from physics-based IR modelling, which could be improved through higher-level electronic-structure methods, anharmonic corrections, improved conformer treatment, condensed-phase modelling and more realistic line shapes.

### 7.2 Descriptor analysis: which molecules benefit from 3D?

To explore which structural features drive the advantage of the 3D-aware PaiNN encoder over the 2D MPNN baseline, we regressed the per-query reciprocal rank difference (PaiNN minus MPNN) against the descriptor panel described in the SI. Fig. 5 reports the PLS coefficients together with two-sided 95% bootstrap confidence intervals from 5000 resamples; an expanded set of targets, including the individual model coefficients, is shown in Fig. S6 and S7.

**Fig. 5:**
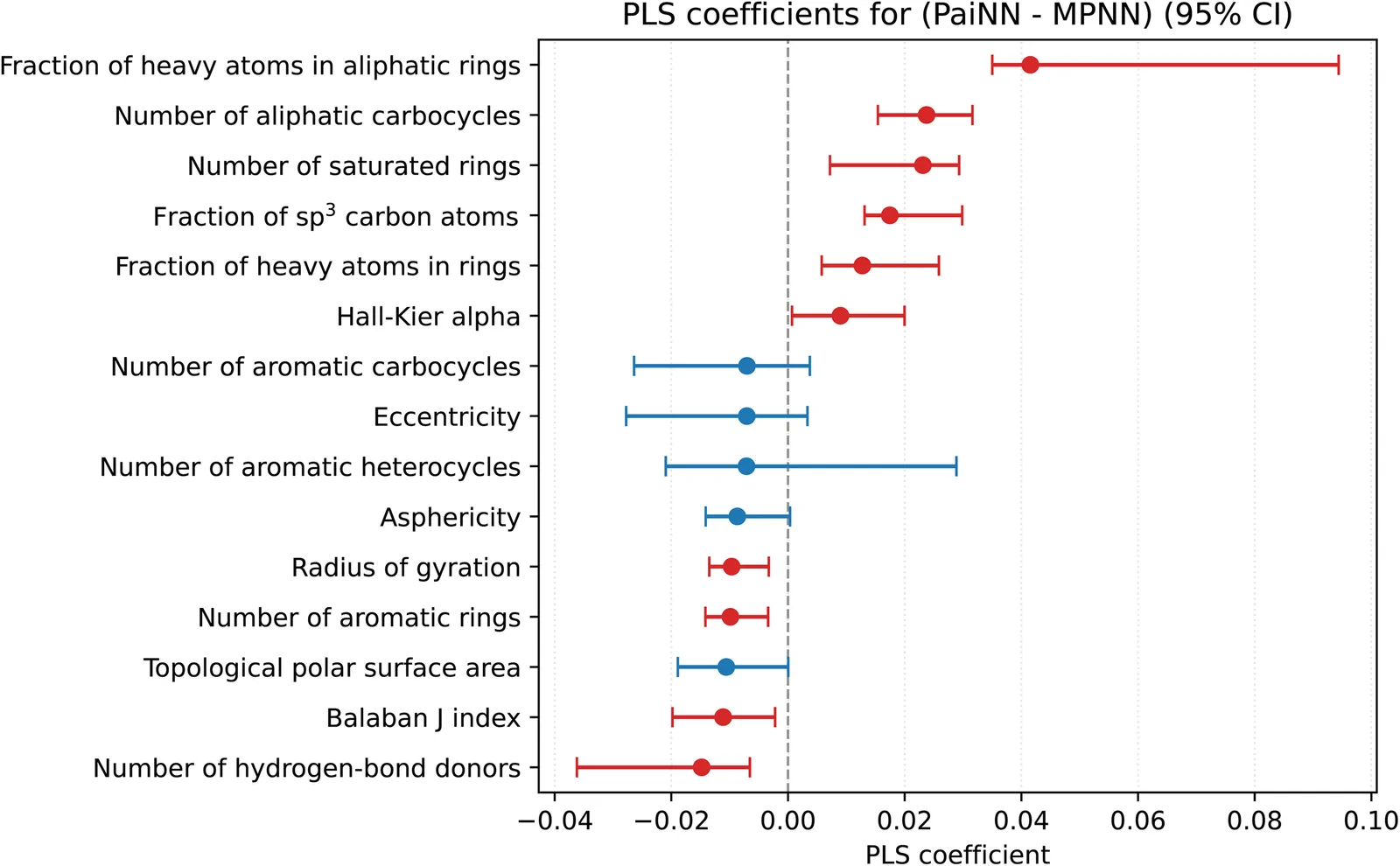
Bootstrapped partial least squares regression coefficients for the PaiNN minus MPNN reciprocal rank target. Points denote the coefficient estimate from the full dataset; horizontal bars show 95% bootstrap confidence intervals over 5000 resamples.

Several descriptors exhibit confidence intervals that exclude zero, indicating directionally stable relationships. The strongest positive coefficients arise from ring-centric features: the fraction of heavy atoms in aliphatic rings (coefficient 0.047; 95% CI [0.040, 0.055]) and in any ring system (0.014; CI [0.008, 0.021]), reinforcing the intuition that conformationally rich, ring-dominated molecules benefit most from a 3D encoder. Conversely, compactness metrics such as the Balaban *J* index (−0.017; CI [−0.023, −0.011]) favour the 2D model when considered individually. While these associations are statistically significant, their magnitudes remain modest, suggesting that coarse descriptors such as the ones we have used may not be sufficient to capture the full complexity of the relevant molecular structures. Taken together, the coefficients suggest that the 3D advantage is distributed across several aspects of ring topology and molecular shape rather than being attributable to a single coarse descriptor. Full numeric summaries are provided in Table S17.

To assess how the retrieval results depend on the number and composition of candidates, we re-scored the fixed test set using restricted candidate sets (Fig. 6). Random candidate sets contain the correct structure plus randomly sampled distractors from the fixed-test candidate pool, whereas nearest-neighbour candidate sets contain the correct structure plus the most similar distractors according to Morgan-fingerprint Tanimoto similarity. As expected, Top-1 accuracy decreases as the candidate set grows. The random candidate-set curves show the effect of pool size alone, while the nearest-Tanimoto curves quantify a harder, chemically enriched setting. At 250 candidates, for example, PaiNN reaches 0.853 Top-1 accuracy on random candidate sets but 0.765 on nearest-Tanimoto candidate sets; the analogous values are 0.781 *versus* 0.664 for MPNN. Thus retrieval difficulty is controlled not only by candidate-set size, but also by the density of structurally similar distractors. These results support the practical use of the retrieval workflow while also clarifying that accuracy estimates for a given library should be interpreted in terms of candidate-set composition. In practical applications, the candidate set would usually not be an arbitrary library of molecules. It would typically be constrained by prior information such as molecular formula, elemental composition, synthetic route, known impurities, reaction starting materials, or complementary spectral data such as NMR or mass spectrometry. The unrestricted fixed-test-set retrieval used here is therefore best viewed as a controlled benchmark for comparing methods, whereas real candidate sets may be either easier, if strongly constrained by prior information, or harder, if enriched with close structural analogues. We therefore also repeated retrieval with every candidate set restricted to structures in the same held-out fold and with the same molecular formula as the query. As expected for these much smaller candidate pools, absolute Top-1 accuracies are higher than in the unrestricted retrieval experiment. PaiNN retained a significant advantage over MPNN in this more constrained setting; full methods and results are reported in SI Section 7.

**Fig. 6:**
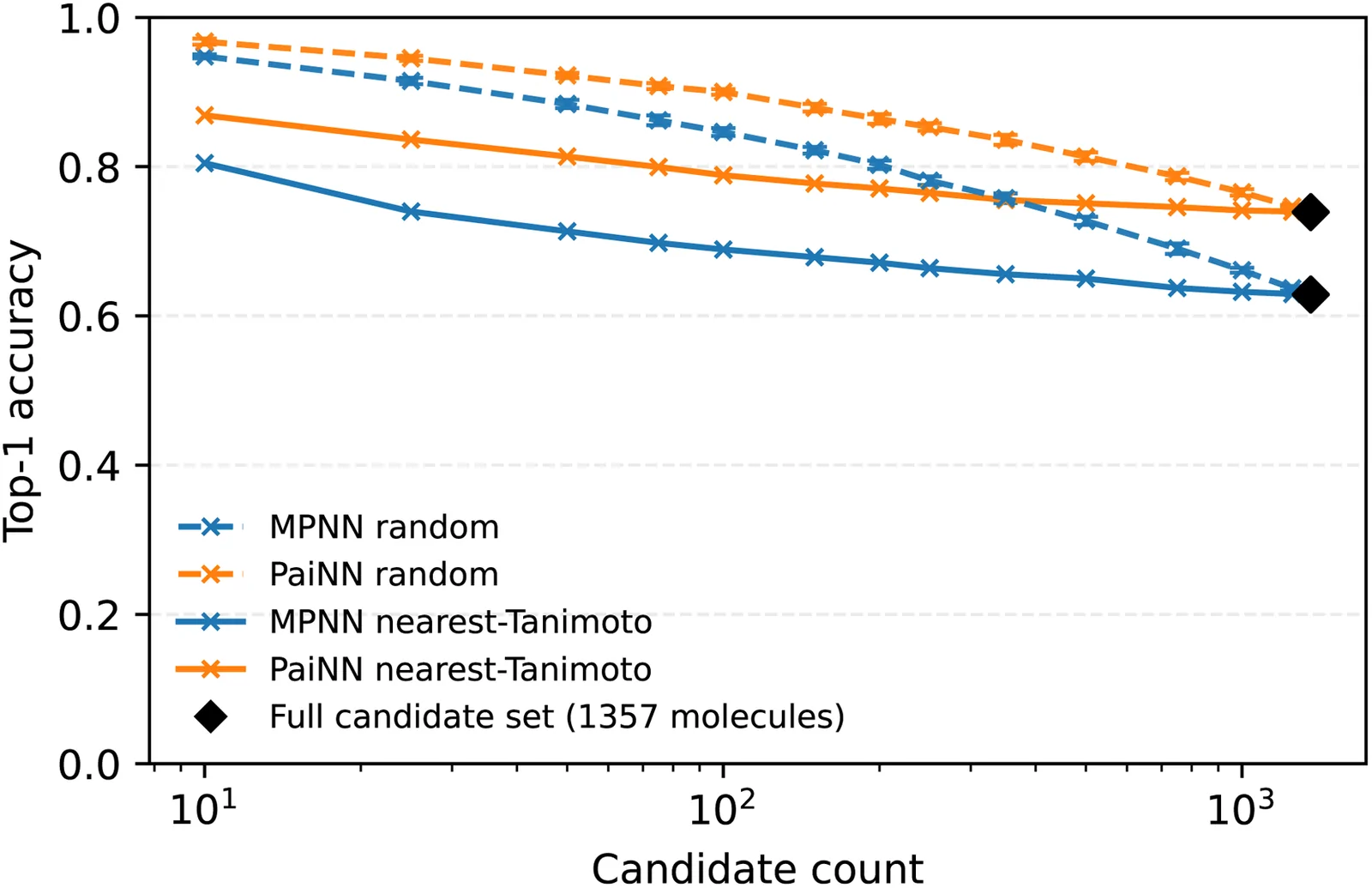
Effect of candidate-set size and composition on Top-1 retrieval accuracy for the fixed 1357-query benchmark (500–3500 cm^−1^, masked cosine). Dashed lines show random candidate sets, which contain the correct structure plus randomly sampled distractors; points and error bars show the mean and sample standard deviation across 20 random draws at each candidate count. Solid lines show nearest-Tanimoto candidate sets, which contain the correct structure plus the most structurally similar distractors by Morgan-fingerprint Tanimoto similarity and are deterministic for a given candidate count. Diamonds mark the full candidate set (1357 molecules).

### 7.3 Structure classification characteristic curve benchmark

We additionally evaluated both encoders using the structure classification characteristic (SCC) curve protocol defined by ref. 43, reproducing the analysis on the published dataset. Fig. 7 shows that the 2D MPNN underperforms the 3D PaiNN across the full SCC curve, consistent with the performance gap seen in our retrieval metrics.

**Fig. 7:**
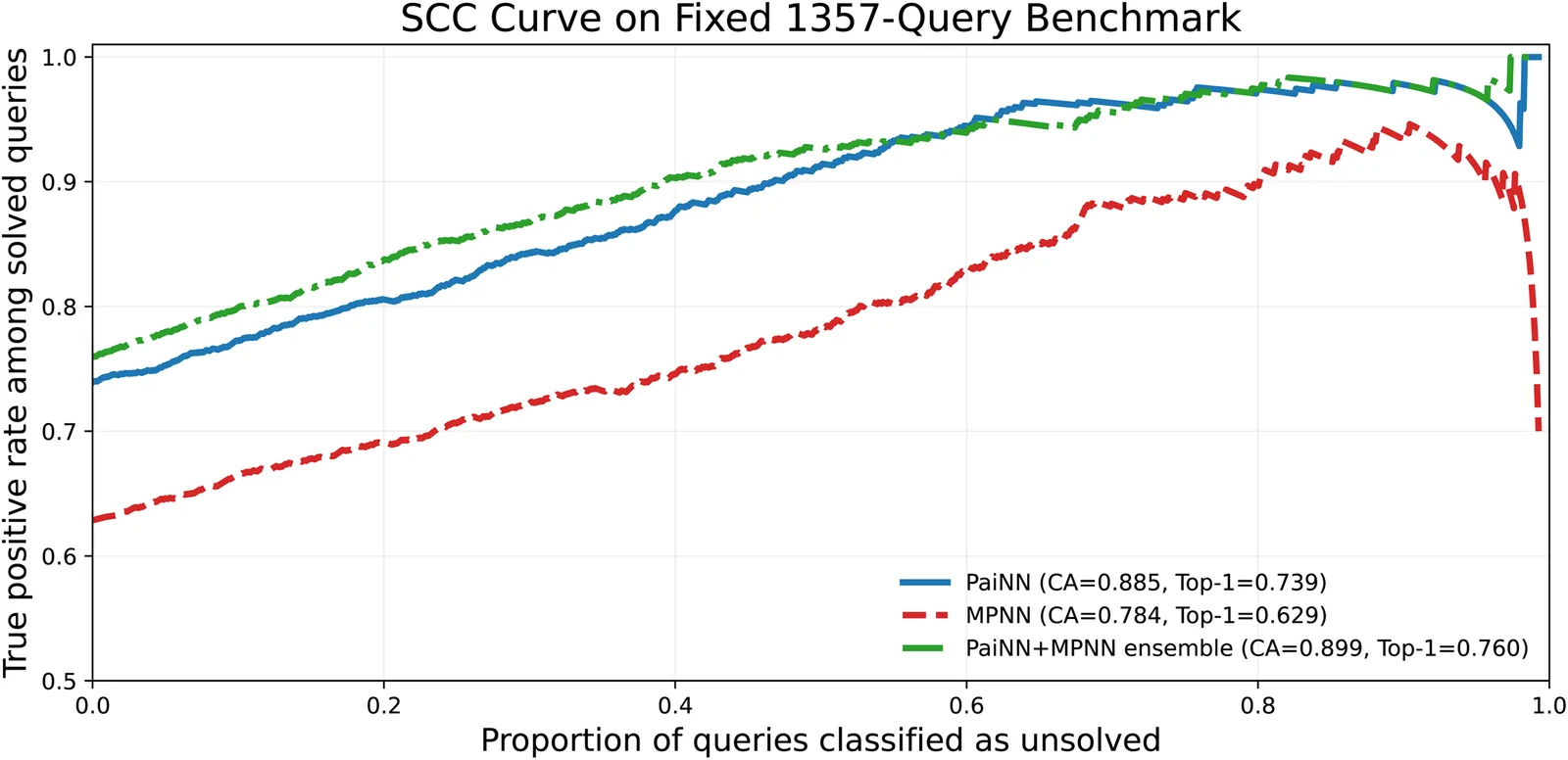
SCC curve reproduced using the dataset and evaluation protocol from ref. 43.

### 7.4 Effect of phase of sample preparation

Fig. 8 shows the mean cosine similarity and Top-1 accuracy for each model, stratified by the phase of sample preparation (gas, liquid, solid). DFT and GFN2-xTB results are included for additional context. All models and calculations perform best on gas-phase spectra, likely due to the reduced complexity of these spectra compared to condensed-phase measurements. For the neural network models, the training data comprised approximately 80% gas-phase spectra, which inevitably results in better performance on gas-phase test spectra. The ensemble of PaiNN and MPNN consistently outperforms the individual models across all phases, and PaiNN outperforms MPNN in all cases.

**Fig. 8:**
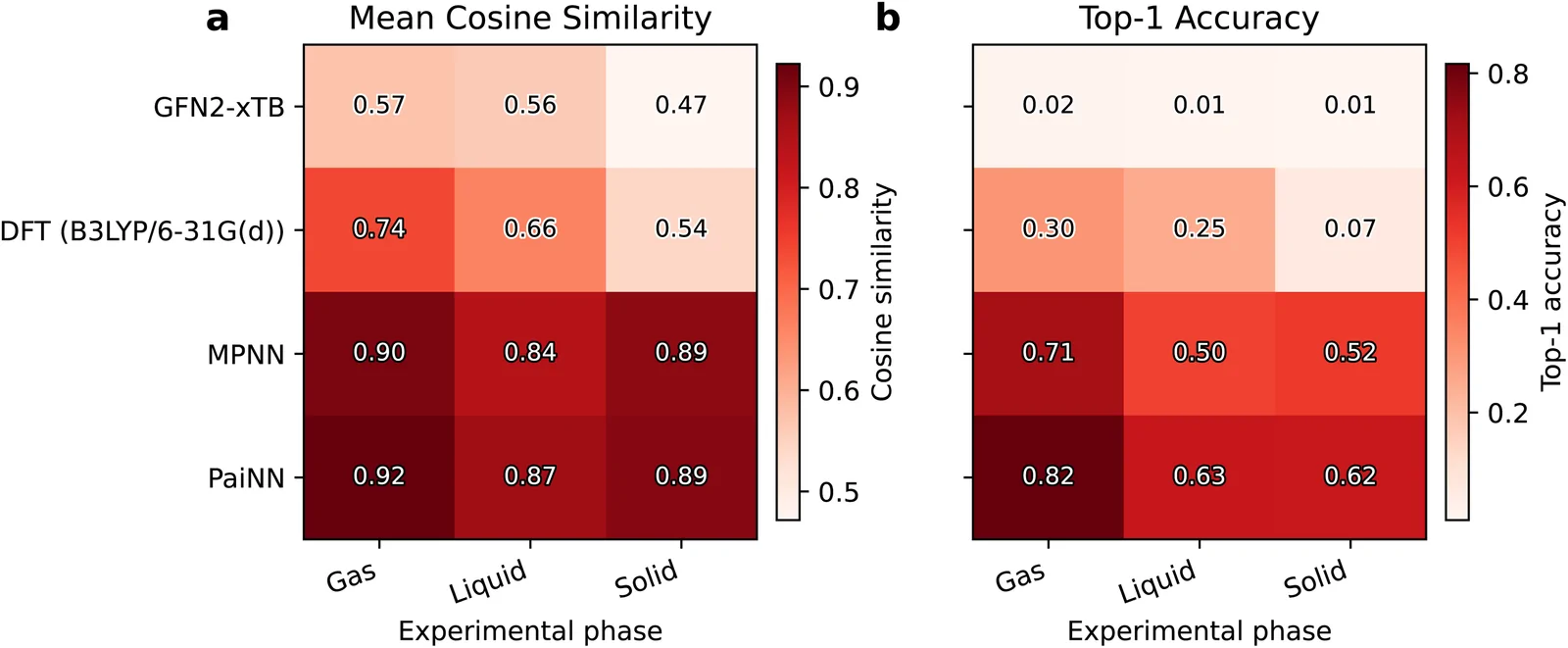
(a) Heat map of mean cosine similarity for each model and experimental phase. (b) Heat map of Top-1 retrieval accuracy for each model and phase. All results are for the 500–3500 cm^−1^ wavenumber range.

### 7.5 Case study

Fig. 9 collects six representative molecules alongside their experimental spectra and the MPNN/PaiNN predictions to illustrate qualitative differences between the encoders. In particular, panel a shows a cyclic hydrocarbon where both models achieve very high cosine similarity (0.974 for MPNN, 0.998 for the 3D model), yet the 3D encoder ranks the structure first while MPNN falls to rank 17. The scarcity of diagnostic peaks means a near-perfect cosine score is insufficient; small shifts in the 2950 cm^−1^ band give the 3D model an advantage. Panel b covers a similarly simple isothiocyanate measured in the gas phase, where both encoders attain Top-1 accuracy (cosine similarity 0.985 for MPNN, 0.998 for the 3D model), demonstrating that straightforward spectra tend to be predicted accurately by either model.

**Fig. 9:**
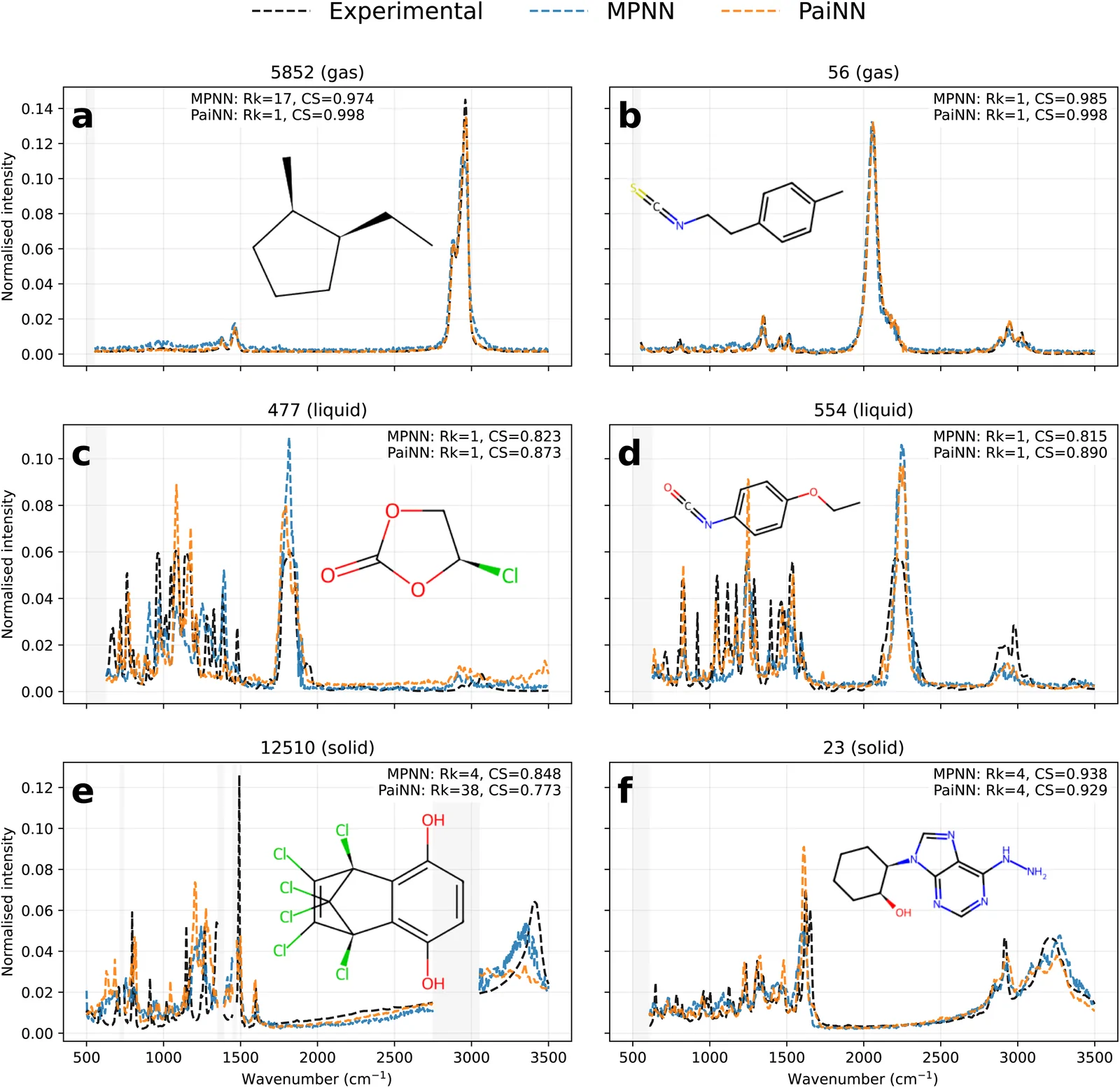
Case study of six representative molecules. For each molecule, the experimental spectrum is shown alongside the predictions from MPNN and PaiNN. The retrieval rank and cosine similarity score for each model is indicated in parentheses. (a and b) Molecules with spectra recorded in the gas phase. (c and d) Molecules with spectra recorded in the liquid phase. (e and f) Molecules with spectra recorded in the solid phase.

Panel c highlights a liquid-phase cyclic carbonate in which the 3D model captures the detailed frequencies and intensities in the fingerprint region more faithfully, although both models achieve rank 1. Panel d provides a similar example where both models reach Top-1 even though the 3D model secures a higher cosine score, demonstrating that retrieval difficulty depends strongly on how many look-alike spectra populate the candidate set.

Panels e and f showcase hydrogen-bond-rich, solid-phase systems with sharp absorption bands. In the example in panel e, the 2D encoder obtains a higher cosine similarity, but neither of the models rank the correct candidate first. In panel f, both models predict the spectrum well, but again neither model succeeds in achieving a top-1 rank.

### 7.6 Computational cost

Fig. 10 shows that MPNN delivers the desired spectra for all 1357 queries within seconds because it operates directly on 2D graphs, whereas PaiNN spends additional time on MMFF geometry preparation yet remains two orders of magnitude faster than the xTB and DFT baselines. The MMFF fine-tuned variant therefore preserves the 3D accuracy gains without reintroducing expensive quantum calculations.

**Fig. 10:**
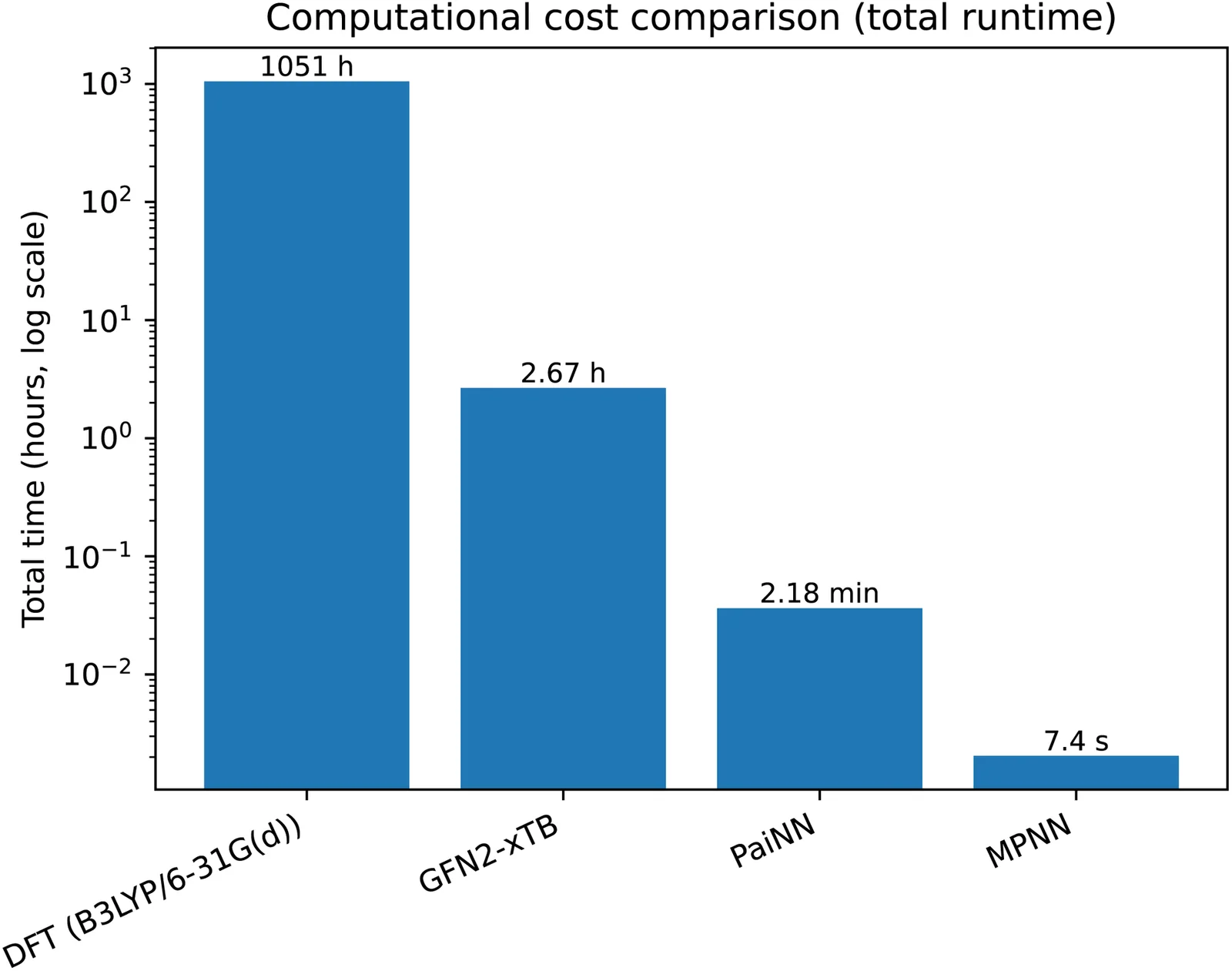
Wall times to process the fixed 1357-molecule test set: MPNN 7.4 s, PaiNN (including MMFF geometry preparation) 2.18 min, GFN2-xTB 2.67 h, and DFT 1051 h on a 16-thread CPU (plus an A100 GPU for neural inference).

## 8 Conclusions

This work re-examines PASGeN, comparing models which use the original 2D encoder (MPNN) with models based on a 3D encoder (PaiNN). We train all models on 13 568 experimental IR spectra using ten-fold cross validation and evaluate structure verification *via* large-scale retrieval against a large number of possible candidates. The ensemble achieves 76.0% Top-1 accuracy compared with 73.9% for PaiNN and 62.9% for MPNN (Table 1). On this benchmark, both models perform substantially above the specific DFT and GFN2-xTB protocols evaluated here. Given the approximations used and the inclusion of condensed-phase experimental spectra, this comparison should not be interpreted as demonstrating general superiority over physics-based IR modelling. Generalisation of the learned models to chemistries beyond those included in the training set is also likely to be limited.

These results highlight the potential of IR spectra as reliable structural fingerprints, as well as showing that incorporation of 3D molecular geometries improves structure verification performance. This makes the approach especially relevant for candidate-ranking problems in which an experimental IR spectrum must distinguish among plausible structures, but full quantum-chemical treatment of every candidate would be too expensive. From a practical standpoint, we recommend the ensemble of PaiNN + MPNN when inference cost allows, because it preserves the advantages of the 3D model while averaging over model-specific errors. Where speed is the primary concern, the 2D MPNN offers a fast and reliable alternative whose predictions still benefit the ensemble. More generally, these results suggest that spectrum prediction frameworks beyond PASGeN may also benefit from 3D geometric information when candidate-ranking structure verification is the target task, provided that the additional conformer-generation cost is acceptable. A further extension would be to augment learned structural embeddings with quantum-derived descriptors, although doing so would introduce additional computational cost and would no longer isolate the effect of changing the molecular encoder.

## Conflicts of interest

RJL and PH are employed by AstraZeneca and own shares in the company. The PhD studentship of JBR is part funded by AstraZeneca.

## Supplementary Material

DD-005-D6DD00281A-s001

## Data Availability

All code required to reproduce the results in this manuscript is available at https://github.com/Goodman-lab/pasgen. Results of GFN2-xTB and DFT calculations, trained model files and prediction outputs are available on Zenodo: https://doi.org/10.5281/zenodo.20069940. Experimental IR spectra are from the NIST Chemistry WebBook. Supplementary information: dataset and preprocessing details, computational methods, model and training details, additional results, significance tests and stratified analyses. See DOI: https://doi.org/10.1039/d6dd00281a.
