# Supplementary material for: Evaluating 2D and 3D graph encoders for infrared-based organic structure verification
Source: Digit Discov. 2026 Aug 19;5(10):4180–90. doi: 10.1039/d6dd00281a (PMC13667908; doi:10.1039/d6dd00281a)
Supplement: Supplementary file 1 [file DD-005-D6DD00281A-s001.pdf]

Supplementary Information (SI) for Digital Discovery.
This journal is © The Royal Society of Chemistry 2026

Supporting Information: Evaluating 2D and 3D Graph Encoders for Infrared-Based Organic Structure Verification

J. Benji Rowlands,[†] Richard J. Lewis,[‡] Peter Howe,[¶] and Jonathan M. Goodman[*,†]

†*Yusuf Hamied Department of Chemistry, University of Cambridge, Cambridge, UK*

‡*Medicinal Chemistry, R&D, AstraZeneca, Gothenburg, Sweden*

¶*Oncology Chemistry, AstraZeneca, Cambridge, UK*

E-mail: jmg11@cam.ac.uk

Contents

1 Dataset Details **3**

1.1 Spectra scraping and normalisation . . . 3

1.2 Structural filtering and conformers . . . 3

1.3 Dataset summary . . . 3

2 Masking of Sample-Preparation Artefacts **5**

3 Computational Methods **5**

4 Models and Hyperparameters **6**

5 Training Details **8**

6 Additional Results **8**

7 Formula-Constrained Retrieval **10**

8 Retrieval Significance Tests **11**

9 Stratified Analyses **15**

10 Regression-on-ranks: Additional Results **16**

References **19**

1 Dataset Details

1.1 Spectra scraping and normalisation

We downloaded around 16 000 digitised IR spectra from the NIST WebBook[1] using a BeautifulSoup script.[2] All traces were converted to absorbance, interpolated onto the $4\,\mathrm{cm}^{-1}$ grid used by PASGeN, and normalised to unit intensity prior to training. We map solution-phase entries onto the liquid category to align with the gas/liquid/solid phase embeddings consumed by the decoder.

1.2 Structural filtering and conformers

For every curated molecule we generated up to ten 3D conformers via RDKit's ETKDG algorithm[3] and optimised them with the MMFF94 force field,[4] and also with GFN2-xTB.[5] Molecules lacking carbon, containing disallowed elements (anything except C, H, N, O, P, S, F, Cl, Br, I, B, or Si), or failing conformer generation were removed. For salts we extracted the organic fragment; entries with multiple distinct organic fragments were discarded. After these filters and the phase annotation step described above we retained 13 927 data points (13 750 unique structures), of which 13 568 include a phase-tagged experimental IR spectrum suitable for learning experiments.

1.3 Dataset summary

Table S1 summarises the curated NIST IR dataset used in this work, including the distribution of sample preparation methods and solvents for solution-phase spectra. Most of the solution-phase spectra are labelled 'Multiple solvents' because the dataset contains lots of spectra recorded in two solvents for different ranges of the spectrum.

Figure S1 shows the molecular weight distribution for the dataset.

Table S1: Composition of the curated NIST IR dataset.

Category	Count	% of dataset
Gas-phase spectra	8,252	59.3
Solution-phase spectra	1,294	9.3
Neat liquid spectra	832	6.0
Solid/film spectra	3,207	23.0
Unspecified phase	342	2.5

Solvent	Count	% of dataset	% of solutions
Multiple solvents	1,052	7.6	81.3
CHCl3	189	1.4	14.6
CCl4	19	0.1	1.5
CS2	17	0.1	1.3
Other solvent	15	0.1	1.2
Unspecified	2	0.0	0.2

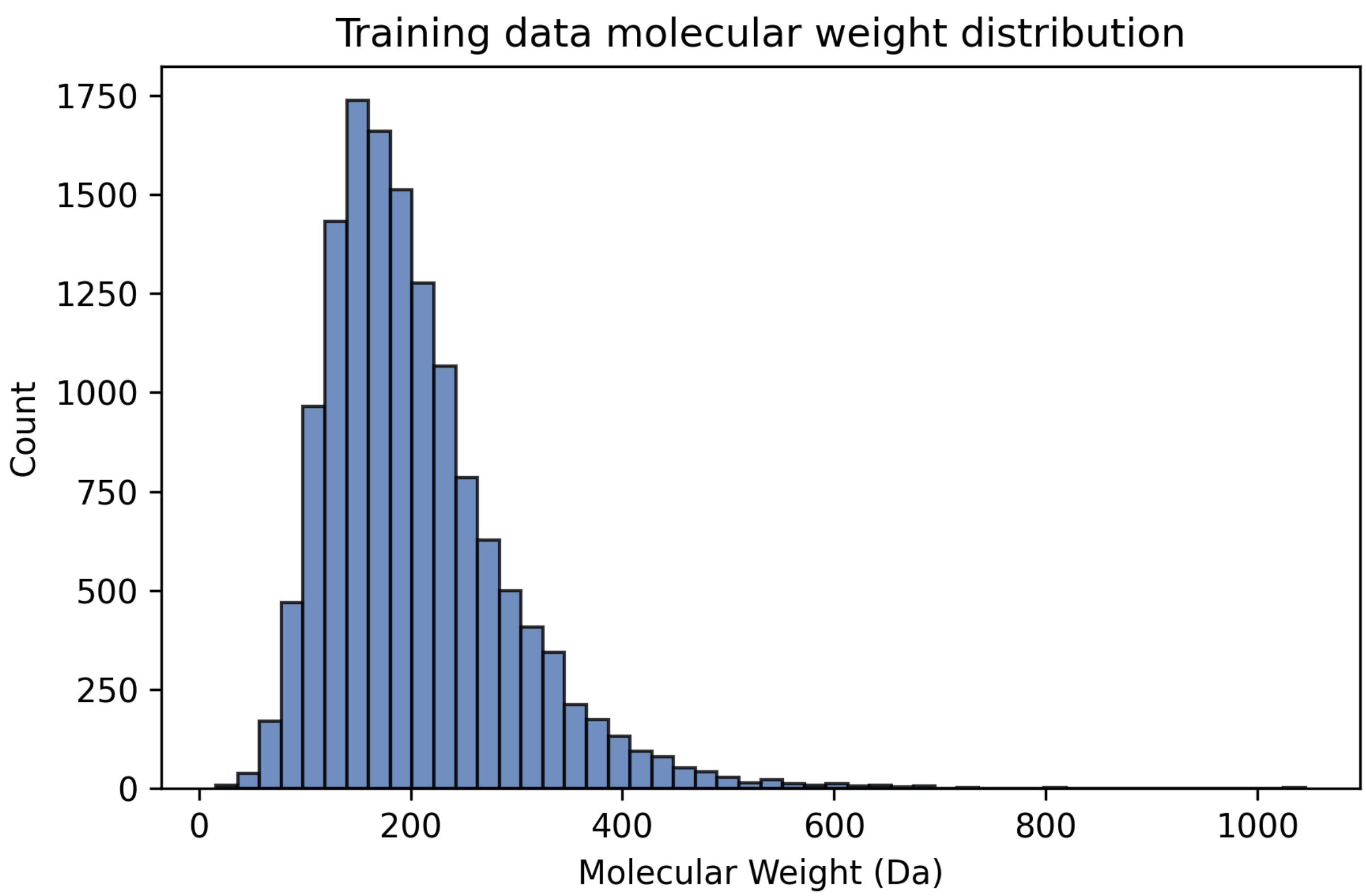

Figure S1: Molecular weight distribution for the curated NIST IR dataset.

2 Masking of Sample-Preparation Artefacts

We parsed each NIST "State" string with a rules-based extractor that recognises named solvents, shorthand identifiers, and explicit wavenumber ranges. Solution phases are treated as "liquid" for consistency with the phase embeddings used by PASGeN. We exclude known solvent bands (for example Nujol, Fluorolube, and CS_2), and we respect any explicit wavenumber limits provided with each spectrum. The resulting boolean mask is applied consistently during training, validation, and evaluation within the chosen wavenumber window. Let $m_i(\tilde{\nu}) \in \{0, 1\}$ denote the mask for spectrum i at wavenumber $\tilde{\nu}$; setting $m_i(\tilde{\nu}) = 0$ removes that region from both the loss and all retrieval metrics. Table S2 reports the solvent-specific masking ranges in ascending order (low–high). When a spectrum's reported evaluation window overlaps only part of a solvent band, we apply masking to the overlapping portion only.

Table S2: Solvent-dependent masked wavenumber ranges. Unless otherwise indicated, values come from the NIST WebBook.[1]

Preparation method	Masked ranges / cm^{-1}
Nujol (mineral-oil mull)[6]	720–740; 1355–1390; 1450–1475; 2750–3050
Fluorolube[7,8]	400–1300
Kel-F (PCTFE)[7,8]	400–1400
Hexachlorobutadiene (HCBD)	400–1300
CS_2	845–875; 1300–1800; 2000–2400
CCl_4 (carbon tetrachloride)	700–850
C_2Cl_4 (tetrachloroethylene)	860–950
$CHCl_3$ (chloroform)	500–1600; 2300–2550; 2850–3300
Heptane	675–800; 1250–1550; 2700–3100

3 Computational Methods

DFT calculations were performed with Gaussian 16 Revision B.01.[9] Initial 3D geometries were generated using the ETKDG algorithm as implemented in RDKit,[3] and pre-optimised with GFN2-xTB.[5] Each conformer was subsequently optimised at the B3LYP/6-31G(d)

level of theory, and harmonic vibrational frequencies were calculated at the same level to confirm that the structures corresponded to minima (no imaginary frequencies). Computed frequencies were scaled by a factor of 0.97.[10] Infrared spectra were obtained by Boltzmann-weighted averaging over the conformational ensemble and broadened using a Lorentzian kernel with a full width at half maximum of 24 cm^{-1} following Pracht.[11]

GFN2-xTB spectra were generated analogously, using Boltzmann-weighted conformer ensembles, with harmonic frequencies scaled by 0.99 as recommended by Pracht.[11]

4 Models and Hyperparameters

The PASGeN decoder employs local windowing, a transformer block, and a lightweight multilayer perceptron (MLP). A learned phase embedding represents gas, liquid, or solid state. For the 2D encoder we use an MPNN with an NNConv layer driven by a learned edge network (rank 32), hidden size of 128, and dropout of 0.1. Each atom is featurised by concatenating the 200-dimensional 'matscholar' elemental embedding, one-hot hybridisation, one-hot formal charge, an aromatic flag, the RDKit degree, and the total hydrogen count. Bonds are represented by a one-hot encoding of the RDKit bond type. Phase encoding is concatenated to edge features, and the pooled molecular embedding is L2-normalised prior to projection. For the 3D encoder we use PaiNN, an equivariant message-passing network with hidden size 128, six interaction blocks, and a 5.0 Å cutoff. Molecular embeddings are obtained by add-pooling followed by L2 normalisation and projection.

Table S3: Key architectural and training hyperparameters.

Component	Setting
Spectrum window size	24 (decoder local windows)
Transformer block	4 heads, head dim 32
Decoder MLP	256 hidden, LeakyReLU + 32 proj + output
Phase embedding	32-dim (joined with atom features via attention pooling)
MPNN hidden size	128 (NNConv), dropout 0.1
MPNN depth	4 message-passing steps
PaiNN	hidden 128, interaction blocks 6, cutoff 5.0 Å
Optimizer	AdamW (lr 5×10^{-4}, weight decay 10^{-4})
Batch size	32
Epochs (max)	500; early stopping: patience 20, min-delta 10^{-4}
Loss	MSE − Pearson correlation (over evaluation range)
Consistency loss	Weight 0.1 (between two sampled conformers)

Table S4: Inputs, featurisations and outputs of the benchmarked methods.

Method	Structural input and featurisation	Architecture and output
MPNN	2D graph; MatScholar element embeddings, hybridisation, formal charge, aromaticity, degree and hydrogen count; bond type.	Four-step NNConv encoder with phase conditioning; PASGeN spectrum decoder.
PaiNN	Atomic identities and coordinates from a force-field-optimised 3D conformer; geometric interactions within a 5.0 Å cutoff.	Six equivariant interaction blocks with phase conditioning; the same PASGeN spectrum decoder.
Original PASGeN	2D molecular graph with the original PASGeN atom and bond representation.	Original message-passing encoder and PASGeN spectrum decoder.
Chemprop-IR	Isomeric SMILES converted to a graph; atomic number, degree, charge, chirality, hydrogen count, hybridisation, aromaticity and mass; bond type, conjugation, ring membership and stereochemistry.	Directed MPNN and feed-forward spectrum predictor; a three-state phase one-hot vector is concatenated to the molecular embedding before prediction.
Contrastive	Molecular graph and experimental spectrum using the published featurisation.	Joint structure–spectrum embedding; direct cosine retrieval rather than spectrum prediction.
B3LYP/6-31G(d)	Boltzmann-weighted 3D conformer ensemble and harmonic frequencies/intensities.	Frequency scaling and constant-width Lorentzian broadening.
GFN2-xTB	Boltzmann-weighted 3D conformer ensemble and harmonic frequencies/intensities.	Frequency scaling and constant-width Lorentzian broadening.

5 Training Details

We optimise all models with AdamW (learning rate 5×10^{-4}, weight decay 10^{-4}, batch size 32) and apply early stopping with a patience of 20 epochs and a minimum improvement of 10^{-4} in the validation loss. The objective is

$$\mathcal{L} = \mathrm{MSE}\big(m \odot y,\ m \odot \hat{y}\big) - \rho\big(m \odot y,\ m \odot \hat{y}\big) + \lambda \left\| \hat{y}^{(a)} - \hat{y}^{(b)} \right\|_2^2, \tag{S1}$$

where ρ is Pearson's correlation, m is the solvent mask from Section 2, and the final term enforces conformer consistency ($\lambda = 0.1$) by penalising the predictions from two independently sampled conformers of the same molecule (the term vanishes when only one conformer is available). The 3D models draw one conformer per step from the precomputed ETKDG ensemble (up to ten members).

6 Additional Results

We additionally include combinatorial retrieval results obtained using the spectral information similarity (SIS) metric instead of cosine similarity. Figures S2–S4 mirror the main text plots under SIS-based ranking: the relative ordering between the stronger 2D/3D learned models is broadly consistent across windows and phases, suggesting that our conclusions are insensitive to the particular spectral similarity metric used to form retrieval rankings.

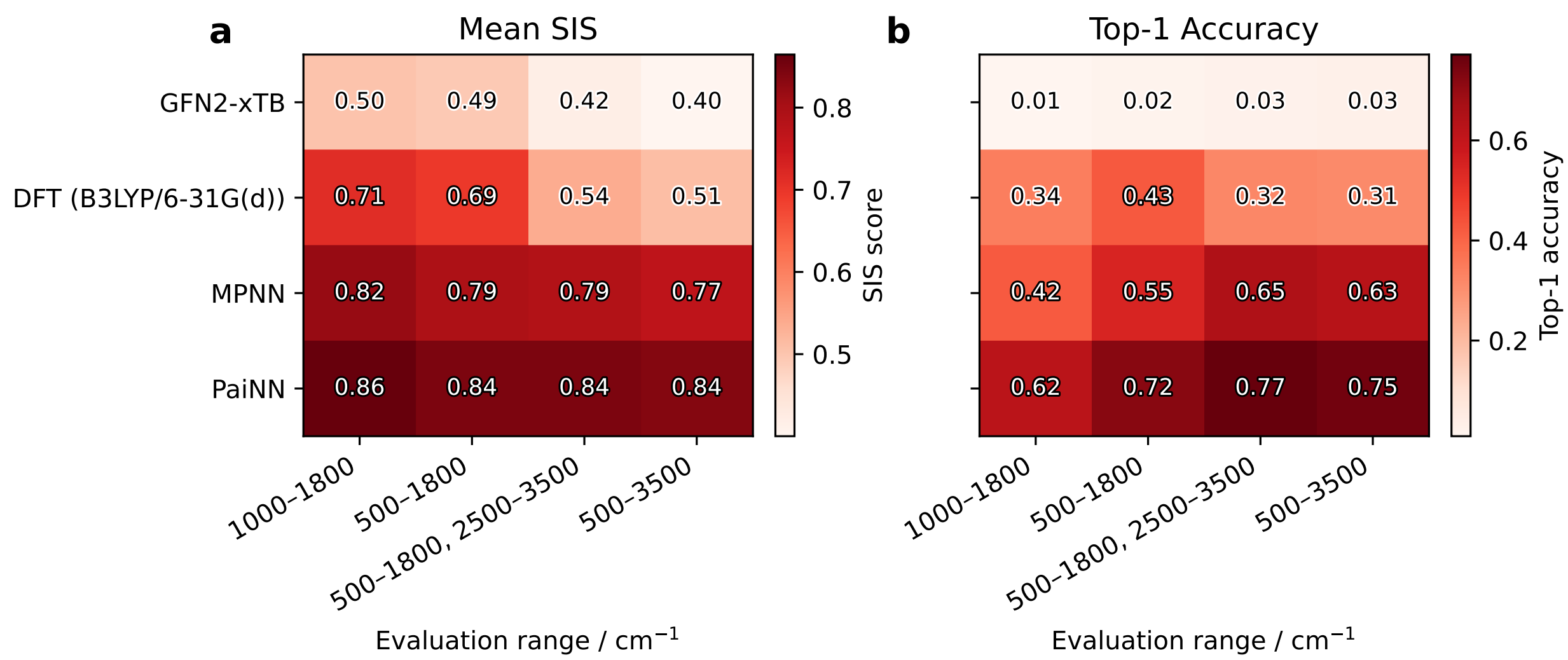

Figure S2: **a** Heat map of mean SIS for each model and wavenumber range. **b** Heat map of Top-1 retrieval accuracy when candidates are ranked by SIS.

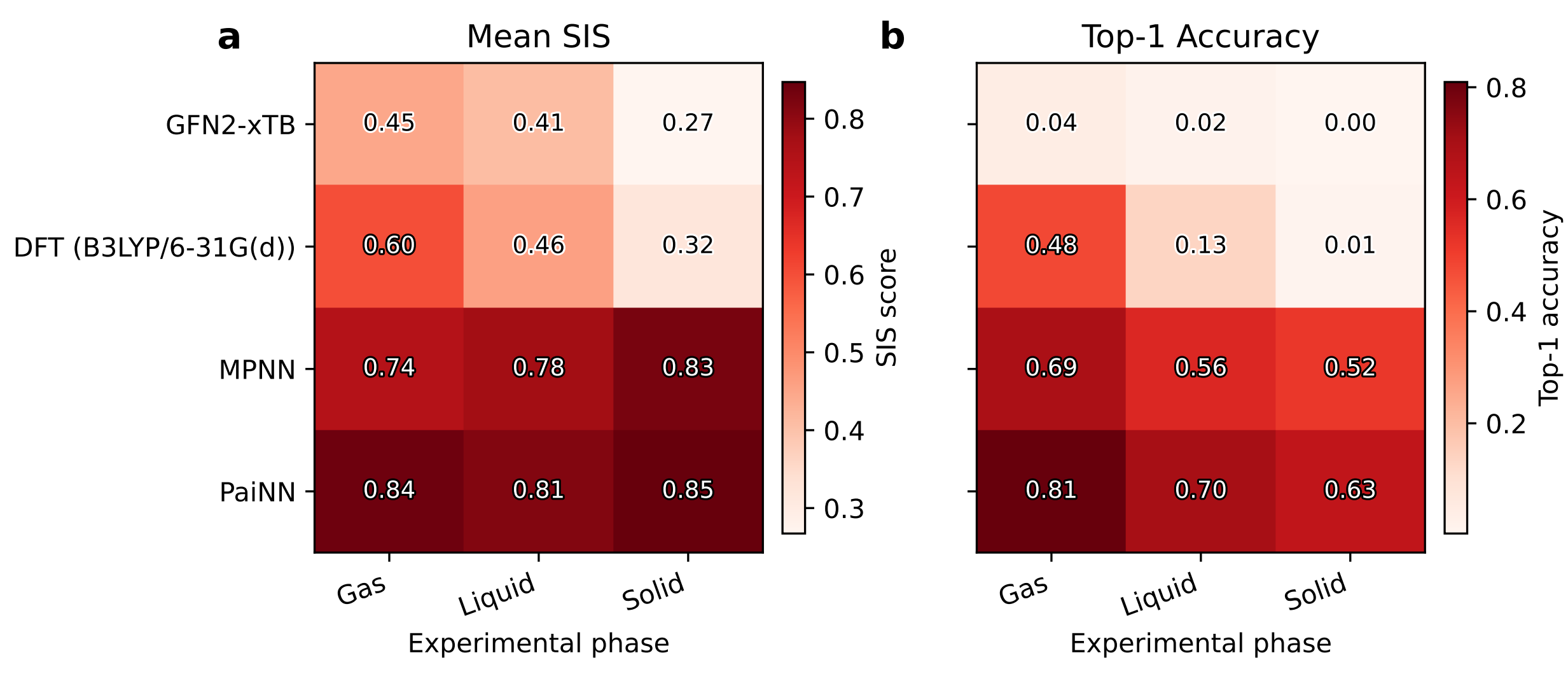

Figure S3: **a** Mean SIS by preparation phase and model. **b** SIS-ranked Top-1 retrieval accuracy by phase.

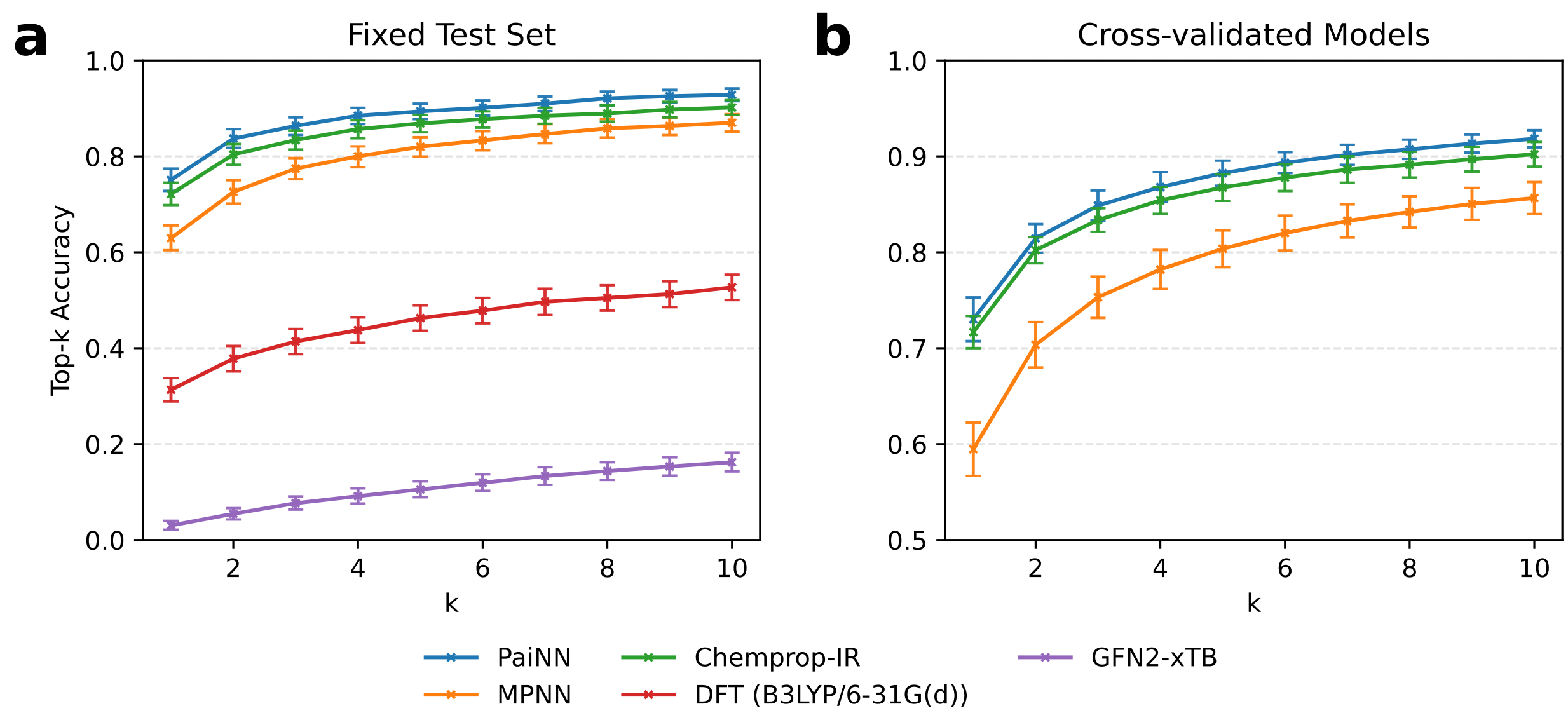

Figure S4: Top-k retrieval accuracy curves when SIS governs the ranking. **a** Results on the fixed 1357-molecule test set (including DFT and GFN2-xTB baselines); error bars denote the bootstrap standard deviation. **b** Results for the neural models over the ten cross-validation folds. Error bars denote the cross-fold standard deviation.

7 Formula-Constrained Retrieval

To model a candidate list constrained by information from mass spectrometry, we repeated the retrieval study with every candidate required to have the same molecular formula as the query. Candidates were restricted to the query's held-out cross-validation fold, and formula groups containing only one record were omitted.

The resulting evaluation set comprises 4084 queries in 1547 formula groups, with a median of 2, mean of 3.09, and maximum of 12 unique candidate structures per query. These pools are much smaller than the full held-out folds used in the unrestricted retrieval experiment, so their absolute Top-1 accuracies are naturally much higher and should not be compared directly with the unrestricted values. This experiment instead tests relative model performance when all candidates have the same molecular formula.

Uncertainty was assessed with 10 000 paired bootstrap resamples at the level of the held-out fold and formula group. PaiNN exceeded MPNN by 3.48 percentage points under cosine ranking (95% CI 2.61–4.36) and by 5.80 points under SIS ranking (95% CI 4.79–6.86); both

Table S5: Top-1 retrieval accuracy when every candidate has the same molecular formula as the query. Results use ten-fold cross-validation predictions over the 500–3500 cm^{-1} window and formula groups containing at least two distinct molecular connectivities.

Model	Cosine Top-1	SIS Top-1
MPNN	90.84%	87.27%
PaiNN	94.32%	93.07%
PaiNN + MPNN	94.42%	92.75%

comparisons gave $p < 0.001$. The ensemble was not significantly different from PaiNN in this restricted analysis.

8 Retrieval Significance Tests

To quantify uncertainty in the headline Top-1 retrieval rates, we bootstrap the per-query success indicators (5000 resamples) for each model and report the resulting 95% confidence intervals alongside standard errors and raw counts (Table S6). Pairwise comparisons between models use exact McNemar tests over the discordant query outcomes (Table S9).

The number of held-out spectra in each cross-validation fold is reported in Table S7. The ten-fold cross-validation experiment over the full dataset is summarised in Table S8, which reports Top-1 accuracy for each held-out fold together with the mean and sample standard deviation for every neural model. The fold-averaged values coincide with the headline retrieval rates reported in the main text.

For completeness we also provide compact-format variants of the same retrieval summaries (Tables S10 and S11).

The same analysis conducted with SIS-based rankings shows nearly identical model orderings with slightly higher absolute accuracies for the 3D methods. Tables S12–S16 tabulate the SIS bootstrap, fold-wise, and McNemar statistics for both MMFF and xTB geometries.

Table S6: Bootstrap Top-1 retrieval accuracy estimates for the 500–3500 cm^{-1} window under cosine-based ranking.

Model	Top-1 ± SE	95% CI	Correct/Total
MPNN	0.623 ± 0.004	[0.614, 0.631]	8448/13566
PaiNN	0.717 ± 0.004	[0.710, 0.725]	9733/13566
Chemprop-IR	0.693 ± 0.004	[0.686, 0.702]	9408/13566
DFT (B3LYP/6-31G(d))	0.241 ± 0.012	[0.218, 0.264]	327/1357
GFN2-xTB	0.015 ± 0.003	[0.010, 0.023]	21/1357

Table S7: Number of held-out spectra in each cross-validation fold.

Fold	1	2	3	4	5	6	7	8	9	10
Held-out spectra	1359	1365	1358	1357	1369	1350	1348	1346	1356	1360

Table S8: Fold-wise Top-1 retrieval accuracy for the ten-fold cross-validation experiments on the 500–3500 cm^{-1} window. Values report Top-1 accuracy per held-out fold; the final column shows the mean and sample standard deviation across folds.

Model	Fold										Mean ± SD
	1	2	3	4	5	6	7	8	9	10	
MPNN	0.648	0.621	0.643	0.618	0.614	0.639	0.676	0.553	0.614	0.601	0.623 ± 0.033
PaiNN	0.715	0.730	0.729	0.731	0.682	0.724	0.732	0.710	0.709	0.712	0.717 ± 0.015
Chemprop-IR	0.685	0.714	0.688	0.704	0.688	0.694	0.704	0.676	0.689	0.693	0.693 ± 0.011

Table S9: Pairwise Top-1 retrieval comparisons with exact McNemar tests and bootstrap confidence intervals for the accuracy differences.

Model A	Model B	ΔTop-1	95% CI	n_{01}	n_{10}	Discordant	McNemar p
MPNN	PaiNN	-0.095	[-0.103, -0.087]	2237	952	3189	< 0.001
MPNN	Chemprop-IR	-0.071	[-0.079, -0.063]	2025	1065	3090	< 0.001
PaiNN	Chemprop-IR	+0.024	[+0.016, +0.031]	1107	1432	2539	< 0.001

Table S10: Bootstrap Top-1 retrieval accuracy estimates in compact format (500–3500 cm^{-1} window).

Model	Top-1	95% CI	Correct/Total
MPNN	0.623	[0.614, 0.631]	8448/13566
PaiNN	0.717	[0.710, 0.725]	9733/13566
Chemprop-IR	0.693	[0.686, 0.702]	9408/13566
DFT (B3LYP/6-31G(d))	0.241	[0.218, 0.264]	327/1357
GFN2-xTB	0.015	[0.010, 0.023]	21/1357

Table S11: Pairwise Top-1 retrieval comparisons in compact format.

Model A	Model B	ΔTop-1	95% CI	n_{01}	n_{10}	Discordant	McNemar p
MPNN	PaiNN	-0.095	[-0.103, -0.087]	2237	952	3189	< 0.001
MPNN	Chemprop-IR	-0.071	[-0.079, -0.063]	2025	1065	3090	< 0.001
PaiNN	Chemprop-IR	+0.024	[+0.016, +0.031]	1107	1432	2539	< 0.001

Table S12: Bootstrap Top-1 retrieval accuracy estimates for the 500–3500 cm^{-1} window under SIS-based ranking.

Model	Top-1 ± SE	95% CI	Correct/Total
MPNN	0.595 ± 0.004	[0.586, 0.603]	8066/13566
PaiNN	0.730 ± 0.004	[0.723, 0.738]	9905/13566
Chemprop-IR	0.717 ± 0.004	[0.709, 0.724]	9724/13566
DFT (B3LYP/6-31G(d))	0.313 ± 0.013	[0.289, 0.338]	425/1357
GFN2-xTB	0.030 ± 0.005	[0.021, 0.040]	41/1357

Table S13: Fold-wise Top-1 retrieval accuracy for the ten-fold cross-validation experiments on the 500–3500 cm^{-1} window when SIS governs the ranking.

Model	Fold										Mean ± SD
	1	2	3	4	5	6	7	8	9	10	
MPNN	0.557	0.632	0.616	0.605	0.546	0.587	0.616	0.577	0.599	0.612	0.595 ± 0.028
PaiNN	0.716	0.744	0.726	0.744	0.690	0.744	0.759	0.727	0.700	0.752	0.730 ± 0.023
Chemprop-IR	0.700	0.721	0.725	0.743	0.702	0.710	0.722	0.689	0.718	0.738	0.717 ± 0.017

Table S14: Pairwise Top-1 retrieval comparisons with exact McNemar tests under SIS-based ranking.

Model A	Model B	ΔTop-1	95% CI	n_{01}	n_{10}	Discordant	McNemar p
MPNN	PaiNN	-0.136	[-0.144, -0.127]	2686	847	3533	< 0.001
MPNN	Chemprop-IR	-0.122	[-0.131, -0.114]	2710	1052	3762	< 0.001
PaiNN	Chemprop-IR	+0.013	[+0.006, +0.021]	1272	1453	2725	< 0.001

Table S15: Bootstrap Top-1 retrieval accuracy estimates in compact format when SIS defines the ranking.

Model	Top-1	95% CI	Correct/Total
MPNN	0.595	[0.586, 0.603]	8066/13566
PaiNN	0.730	[0.723, 0.738]	9905/13566
Chemprop-IR	0.717	[0.709, 0.724]	9724/13566
DFT (B3LYP/6-31G(d))	0.313	[0.289, 0.338]	425/1357
GFN2-xTB	0.030	[0.021, 0.040]	41/1357

Table S16: Pairwise Top-1 retrieval comparisons in compact format under SIS-based ranking.

Model A	Model B	ΔTop-1	95% CI	n_{01}	n_{10}	Discordant	McNemar p
MPNN	PaiNN	-0.136	[-0.144, -0.127]	2686	847	3533	< 0.001
MPNN	Chemprop-IR	-0.122	[-0.131, -0.114]	2710	1052	3762	< 0.001
PaiNN	Chemprop-IR	+0.013	[+0.006, +0.021]	1272	1453	2725	< 0.001

9 Stratified Analyses

To see how structural features line up with retrieval performance, we split queries by individual descriptors into "low" and "high" subsets using 20% percentile cuts and report Top-1 accuracy with 95% bootstrap confidence intervals (5000 resamples). The subsets are univariate and so do not account for confounding variables. In addition, highly skewed descriptors can leave one subset much larger than the other. The plots should therefore be read as descriptive summaries that demonstrate the qualitative trends from the main text: molecules with a high fraction of sp^3 atoms or atoms in aliphatic rings give PaiNN a bigger advantage, whereas compactness descriptors such as Balaban J or a large number of hydrogen bond donors narrow the gap and make the 2D MPNN more competitive.

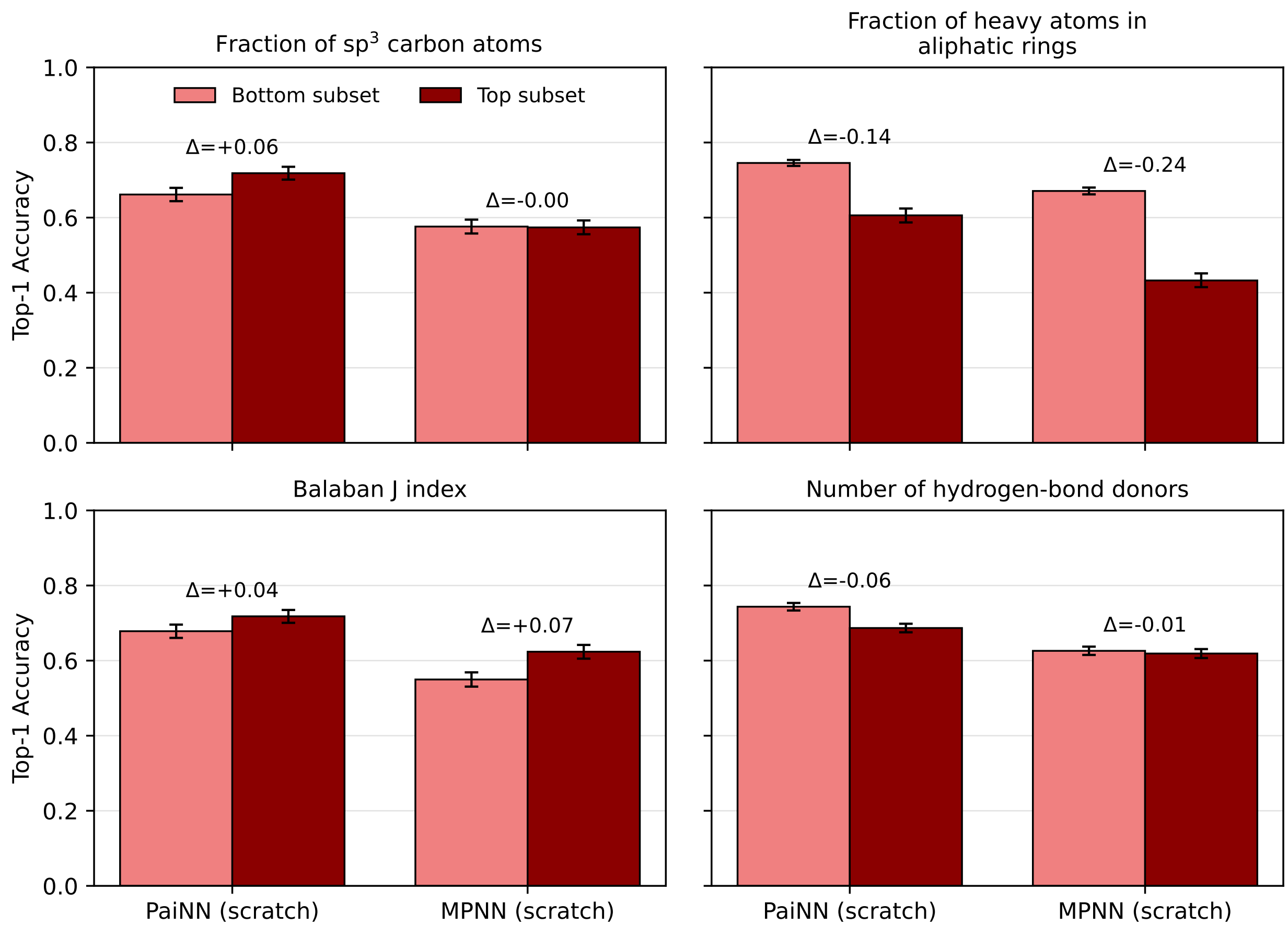

Figure S5: Cross-validated Top-1 accuracy (500–3500 cm^{-1}, cosine-ranked) for descriptor-defined "low" (salmon) and "high" (dark red) subsets, obtained via 20% percentile splits (note that skewed descriptors lead to unequal subset sizes). Bars show PaiNN (left) and MPNN (right); error bars denote 95% per-query bootstrap confidence intervals over 5 000 resamples.

10 Regression-on-ranks: Additional Results

Table S17 reports the leading coefficients for the difference target, while Figures S6 and S7 summarise the individual encoder fits. Despite several statistically robust associations, most notably ring fractions in favour of PaiNN and compactness metrics in favour of MPNN, the effect sizes are fairly small.

Table S17: Bootstrapped PLS coefficients for the 3D-model minus MPNN reciprocal rank target. Coefficients are means over 5000 resamples with 95% confidence intervals and two-sided bootstrap p-values.

Feature	Coefficient	95% CI	p
Fraction of heavy atoms in aliphatic rings	+0.042	[+0.035, +0.094]	0.0e+00
Number of aliphatic carbocycles	+0.024	[+0.015, +0.032]	0.0e+00
Number of saturated rings	+0.023	[+0.007, +0.029]	0.004
Fraction of sp^3 carbon atoms	+0.017	[+0.013, +0.030]	0.0e+00
Fraction of heavy atoms in rings	+0.013	[+0.006, +0.026]	4.0e-04
Hall-Kier alpha	+0.009	[+0.001, +0.020]	0.034
Number of aromatic carbocycles	-0.007	[-0.026, +0.004]	0.226
Eccentricity	-0.007	[-0.028, +0.003]	0.165
Number of aromatic heterocycles	-0.007	[-0.021, +0.029]	0.671
Asphericity	-0.009	[-0.014, +0.000]	0.059
Radius of gyration	-0.010	[-0.014, -0.003]	0.010
Number of aromatic rings	-0.010	[-0.014, -0.003]	0.004
Topological polar surface area	-0.011	[-0.019, +0.000]	0.051
Balaban J index	-0.011	[-0.020, -0.002]	0.021
Number of hydrogen-bond donors	-0.015	[-0.036, -0.007]	0.004

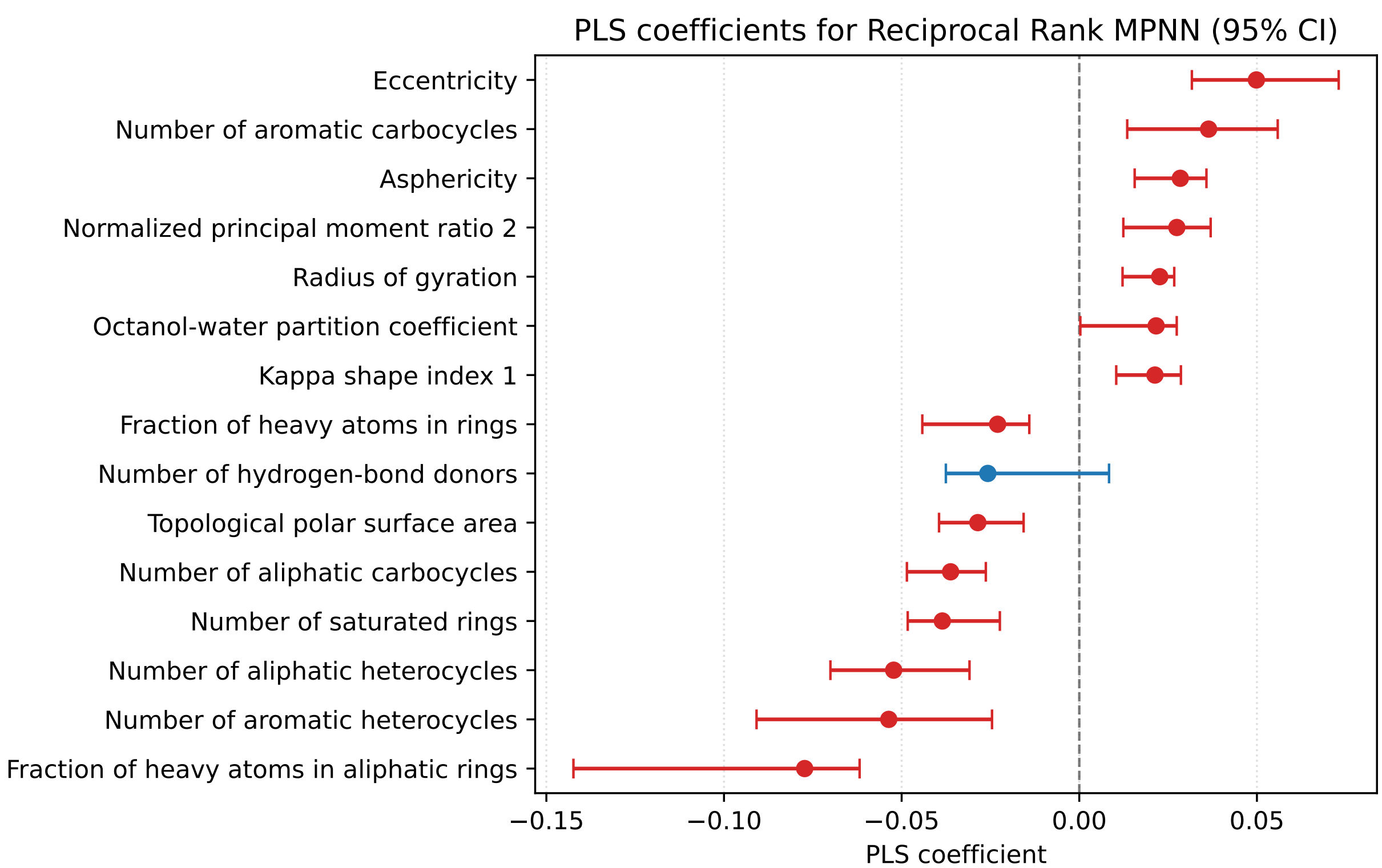

Figure S6: Bootstrapped PLS coefficients for the MPNN reciprocal rank target. Points denote full-sample coefficient estimates; horizontal bars indicate 95% confidence intervals across 5000 bootstrap resamples.

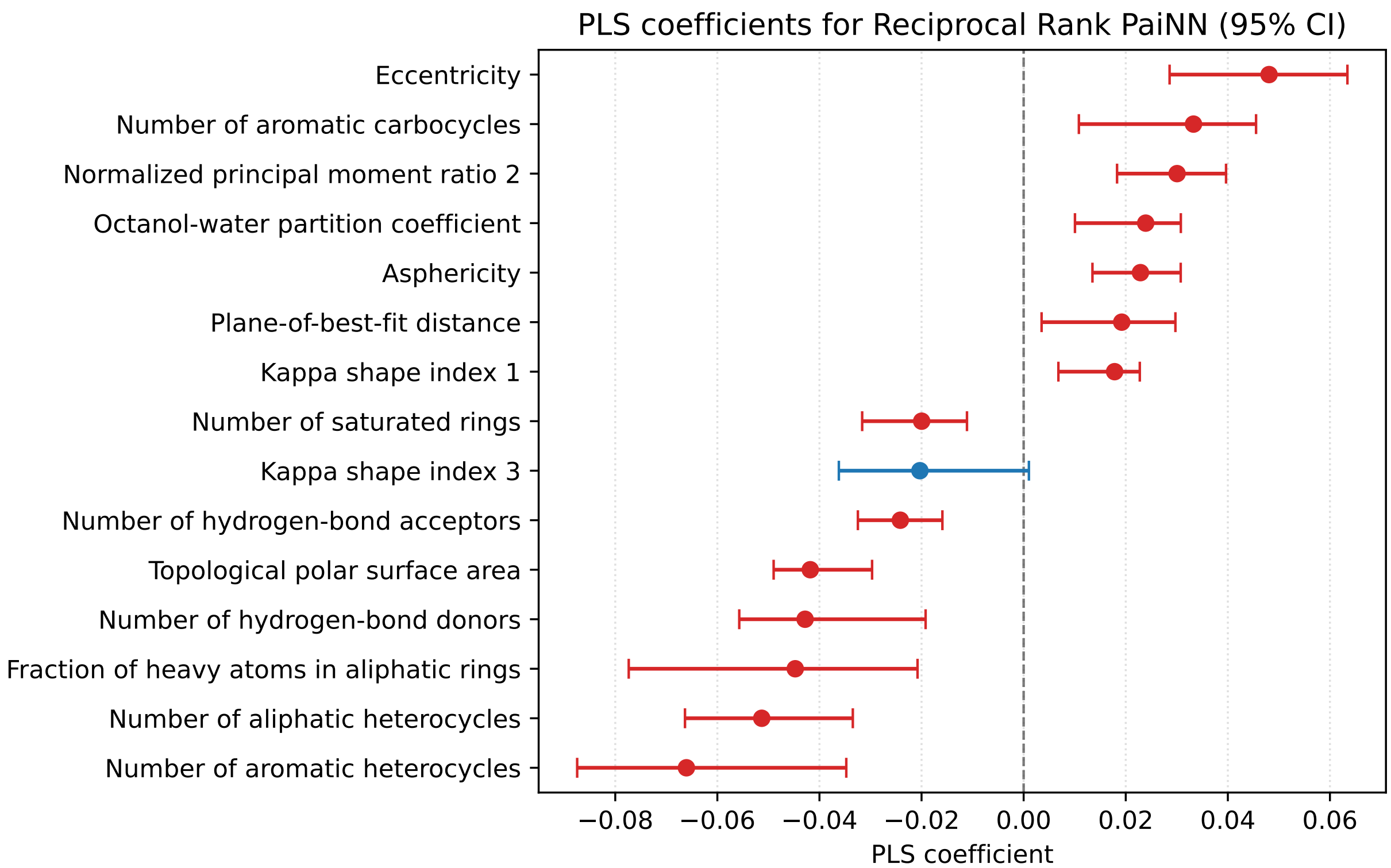

Figure S7: Bootstrapped PLS coefficients for the 3D-model reciprocal rank target. Points denote full-sample coefficient estimates; horizontal bars indicate 95% confidence intervals across 5000 bootstrap resamples.

References

(1) NIST Mass Spectrometry Data Center In *NIST Chemistry WebBook, NIST Standard Reference Database Number 69*; Lindstrom, P. J., Mallard, W. G., Eds.; National Institute of Standards and Technology: Gaithersburg, MD.

(2) Saquer, N.; Iqbal, R.; Ellis, J. D.; Yoshimatsu, K. Infrared Spectra Prediction Using Attention-Based Graph Neural Networks. *Digital Discovery* **2024**, *3*, 602–609.

(3) Riniker, S.; Landrum, G. A. Better Informed Distance Geometry: Using What We Know To Improve Conformation Generation. *J. Chem. Inf. Model.* **2015**, *55*, 2562–2574.

(4) Halgren, T. A. MMFF VI. MMFF94s Option for Energy Minimization Studies. *J. Comput. Chem.* **1999**, *20*, 720–729.

(5) Bannwarth, C.; Ehlert, S.; Grimme, S. GFN2-xTB—An Accurate and Broadly Parametrized Self-Consistent Tight-Binding Quantum Chemical Method with Multipole Electrostatics and Density-Dependent Dispersion Contributions. *J. Chem. Theory Comput.* **2019**, *15*, 1652–1671.

(6) Iorio, F. B. R. D.; Liberatore, A. M. A.; Koh, I. H. J.; Otani, C.; Camilo, F. F. Ozonated Mineral Oil: Preparation, Characterization and Evaluation of the Microbicidal Activity. *Ozone Sci. Eng.* **2016**, *38*, 253–260.

(7) Kawano, Y.; Araujo, S. C. D. Raman and Infrared Spectra of Polychlorotrifluoroethylene. *J. Braz. Chem. Soc.* **1996**, *7*, 491–496.

(8) Liang, C. Y.; Krimm, S. Infrared Spectra of High Polymers. III. Polytetrafluoroethylene and Polychlorotrifluoroethylene. *J. Chem. Phys.* **1956**, *25*, 563–571.

(9) Frisch, M. J. et al. Gaussian 16, Revision B.01. Gaussian, Inc., 2016.

(10) Alecu, I. M.; Zheng, J.; Zhao, Y.; Truhlar, D. G. Computational Thermochemistry: Scale Factor Databases and Scale Factors for Vibrational Frequencies Obtained from Electronic Model Chemistries. *J. Chem. Theory Comput.* **2010**, *6*, 2872–2887.

(11) Pracht, P.; Grant, D. F.; Grimme, S. Comprehensive Assessment of GFN Tight-Binding and Composite Density Functional Theory Methods for Calculating Gas-Phase Infrared Spectra. *J. Chem. Theory Comput.* **2020**, *16*, 7044–7060.
